# Functional associations of evolutionarily recent human genes exhibit sensitivity to the 3D genome landscape and disease

**DOI:** 10.1101/2024.03.17.585403

**Published:** 2024-03-17

**Authors:** Katherine Fleck, Victor Luria, Nitanta Garag, Amir Karger, Trevor Hunter, Daniel Marten, William Phu, Kee-Myoung Nam, Nenad Sestan, Anne H. O’Donnell-Luria, Jelena Erceg

**Affiliations:** 1 Department of Molecular and Cell Biology, University of Connecticut, Storrs, CT 06269; 2 Institute for Systems Genomics, University of Connecticut, Storrs, CT 06269; 3 Department of Neuroscience, Yale School of Medicine, New Haven, CT 06510; 4 Division of Genetics and Genomics, Boston Children’s Hospital, Boston, MA 02115; 5 Program in Medical and Population Genetics, Broad Institute of Harvard and MIT, Cambridge, MA 02142; 6 Department of Systems Biology, Harvard Medical School, Boston, MA 02115; 7 IT-Research Computing, Harvard Medical School, Boston, MA 02115; 8 Department of Molecular, Cellular and Developmental Biology, Yale University, New Haven, CT 06510; 9 Department of Pediatrics, Harvard Medical School, Boston, MA 02115; 10 Department of Genetics and Genome Sciences, University of Connecticut Health Center, Farmington, CT 06030

**Keywords:** 3D genome organization, gene evolution, human accelerated regions (HARs), gene function/ontology, cell type-specific processes

## Abstract

Genome organization is intricately tied to regulating genes and associated cell fate decisions. In this study, we examine the positioning and functional significance of human genes, grouped by their evolutionary age, within the 3D organization of the genome. We reveal that genes of different evolutionary origin have distinct positioning relationships with both domains and loop anchors, and remarkably consistent relationships with boundaries across cell types. While the functional associations of each group of genes are primarily cell type-specific, such associations of conserved genes maintain greater stability across 3D genomic features and disease than recently evolved genes. Furthermore, the expression of these genes across various tissues follows an evolutionary progression, such that RNA levels increase from young genes to ancient genes. Thus, the distinct relationships of gene evolutionary age, function, and positioning within 3D genomic features contribute to tissue-specific gene regulation in development and disease.

## Introduction

The genome is intricately folded and regulated in the nucleus, which may contribute to cell type diversity. Recent technological advances in mapping of chromosomal interactions and single-cell imaging have provided insights into the organization of chromatin at various levels (1–4). Domains (also termed topologically associating domains or TADs) with increased chromatin interactions may be insulated from neighboring regions by boundaries with decreased chromatin interactions (5–8). Formation of these domains is often mediated by extrusion of chromatin loops, which dynamically link distal anchor points (6, 9–12). However, the interplay between genome structure and function is still unclear (13–16). For instance, while some chromosomal disruptions and perturbations of regulators involved in genome folding may not lead to substantial effects on gene regulation, other structural disruptions at specific loci may affect contacts between regulatory regions, leading to cancer and congenital limb malformations (13, 14, 17–22). Moreover, communication between regulatory regions associated with gene activation is facilitated by direct chromatin looping or other hub-like structures (6, 13, 14, 23–34). Having proper regulatory interactions, including those between enhancers and promoters, is key for cell type-specific gene activity.

Understanding how the genome is organized and the extent to which its organization is conserved may illuminate the functional significance of genomic regions. For instance, perfectly conserved sequences between distantly related species that diverged 90 to 300 million years ago, named ultraconserved elements (UCEs) (35–38), have non-random relationships to 3D genome organization (39). Specifically, UCEs are enriched in domains and associated with renal and neuronal processes when present in domains that are common to many cell types (39). Clusters of conserved noncoding elements (CNEs) around developmental genes, with 70–90% CNE conservation level, are also highly concordant with domains (40). As for human accelerated regions (HARs), sequences conserved in vertebrates but fast-evolving in the human lineage (41–49), they are associated with domains that have human-specific structural variants, suggesting potential rewiring of contacts between regulatory regions (50). Moreover, structural variants and mutations affecting conserved and fast-evolving regions are often related to diseases and neurodevelopmental disorders (37, 46, 51–55).

Protein-coding genes also experience varying levels of conservation, from extremely high sequence conservation across evolutionary taxa to *de novo* evolution in specific lineages. To understand how gene expression and function emerge and change with time, genes of different evolutionary ages can be compared once the minimal evolutionary age of each gene is evaluated (56–58). Novel protein-coding genes originating by non-copying mechanisms (*de novo* from intergenic regions; from long non-coding RNAs that become coding; or by overprinting, that is, translating an existing mRNA in a different frame) have been discovered since 2006 by studying naturally occurring gene variants, through mutagenesis screens and by proteogenomics in species ranging from plants to humans (56, 59–64). Such novel genes are expressed in different tissues (65, 66), have function (59, 67–71) and, in contrast to new genes arising from copying mechanisms (gene or genome duplication; retrotransposition; exon shuffling; or pseudogene resurrection), can have novel protein domains (70–72). The process of gene birth and death is continuous, and some genes are specific to particular lineages (73).

Here, we asked how the positionings of genes of different evolutionary ages in the 3D genome landscape relate to their expression and functional significance. Interestingly, while genes of different evolutionary ages have distinct relationships with domains and loop anchors, their relationships with boundaries are consistent across cell types. Furthermore, evolutionarily older genes maintain stable functional associations regardless of genomic positioning and are expressed at higher levels than more recently evolved genes, which exhibit functional associations that are sensitive to the positioning of these genes within the 3D genome organization and disease. Together, the distinct 3D genomic positioning of genes as a function of their evolutionary age may be related to the regulation of tissue-specific programs in development and disease.

## Results

### Regions of varying sequence conservation have distinct relationships with 3D genomic features across cell types

**Fig. 1.**
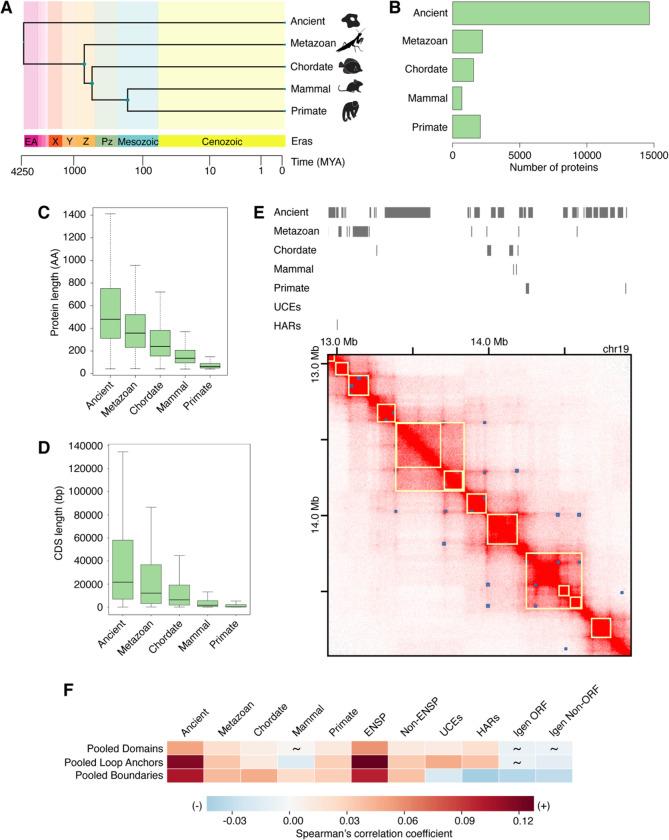
Genomic regions of varying sequence conservation have distinct relationships with features of 3D genome organization. (*A*) Human genes assigned an evolutionary age by phylogenomics and phylostratigraphy. Representative species used with TimeTree (136) to build a timetree: *Escherichia coli* (ancient), *Amphimedon queenslandica* (metazoan), *Branchiostoma floridae* (chordate), *Ornithorhynchus anatinus* (mammal), and *Homo sapiens* (primate). (*B*) Number of single-isoform human protein-coding genes in every evolutionary era (ancient, metazoan, chordate, mammal, primate). Most genes are ancient. (*C*) Protein length is indicated in number of amino acids (AA) and is largest for ancient genes. (*D*) The length of coding sequences (CDS) is indicated as the number of base pairs (bp) and is largest for ancient genes. (*E*) Visualization of example genomic regions of varying sequence conservation with the GM12878 Hi-C dataset (6) using the Juicebox tool (82). Domains (yellow squares) and peaks indicating loop presence (blue squares), as annotated in Juicebox. (*F*) Pooled domains, loop anchors, and boundaries are significantly positively correlated with era genes (1.08×10^−172^ ≤ p ≤ 0.022), except for mammal genes in pooled domains and loop anchors (6.03×10^−5^ ≤ p ≤ 0.654). UCEs and HARs are significantly positively correlated with pooled domains and loop anchors, but negatively correlated with pooled boundaries (1.34×10^−29^ ≤ p ≤ 0.017). Spearman correlation analysis was performed by partitioning the genome into 50-kb bins. Spearman correlation coefficients are indicated by a heatmap. Non-significant p values are depicted by tildes. Control datasets include ENSP genes, Non-ENSP genes, Igen ORFs, and Igen Non-ORFs.

To understand the relationship between gene age and features of 3D genome organization, we built a dataset with all human protein-coding genes and control intergenic sequences. First, we evaluated the evolutionary age of all human protein-coding genes (Fig. 1A and Fig. S1). Of these 21,436 single-isoform genes (only the oldest and longest protein isoform was kept), most (19,334) are annotated in Ensembl 89 (Fig. S1A). Additionally, 2,102 unannotated genes with translation evidence from proteogenomics studies (74–77) were also used (Fig. S1B). We estimated gene evolutionary age by phylostratigraphy. This method estimates the minimal evolutionary age of a gene by determining the most distant species in which a protein sequence sufficiently similar to the query sequence is detected, and inferring that the gene must be at least as ancient as the common ancestor of the query species and the most distant species (57, 78, 79). Thus, a human gene for which a sufficiently similar sequence exists in bacteria is Ancient (14,845 genes); a human gene also present in sponges but not in more distant species is specific to Metazoan (2253 genes); a human gene also present in lancelets is specific to Chordates (1584 genes); a human gene also present in platypus but not in more distant species is specific to Mammals (685 genes); a human gene also present in lemurs is specific to Primates (2069 genes) (Fig. 1B). We refer to these five categories (ancient, metazoan, chordate, mammal, primate) based on the evolutionary age of genes as *eras* and genes within them as *era genes*. As controls for human protein-coding genes of varying evolutionary age, we used intergenic open reading frames (Igen ORFs, 3924 sequences) that are potential protein-coding sequences with no known evidence of transcription or translation. Additionally, we used human intergenic sequences that cannot encode proteins (Igen Non-ORFs, 3892 sequences) and are matched in length to Igen ORFs (Table S1). We find that there is an evolutionary progression from short young genes to long ancient genes (Fig. 1 C and D), as noted previously in multiple eukaryotic species (56, 57, 80). Interestingly, the youngest genes are very similar in size to control Igen ORFs (Fig. S1 C and D), suggesting young genes are picked at random from the myriad of ORFs present in the vast intergenic parts of the human genome.

Second, we included two datasets with different levels of sequence conservation. The first dataset is comprised of UCEs, genomic regions at least 200 base pairs (bp) in length that share 100% sequence identity between reference genomes of distantly related species. Comparisons between human, mouse, and rat; or human, dog, and mouse; or human and chicken genomes identified 896 UCEs in total (39, 53, 81). The second dataset is comprised of >3,100 HARs corresponding to sequences that are conserved in vertebrates but are rapidly evolving in the human genome (41–49).

We began by visualizing the placement of era genes, UCEs, and HARs in the 3D genome landscape as illustrated with an example region of the GM12878 genome (6, 82) (Fig. 1E). Then, to globally assess how genomic regions of varying sequence conservation relate to features of 3D genome organization such as domains, loop anchors, and boundaries, we compiled Hi-C datasets from various published studies encompassing a wide range of cell types (Table S1) (5, 6, 19, 83–93). Due to the variation in methodology between these studies (e.g. Hi-C protocol, sequencing depth, quantity of input material), we considered each dataset individually as well as grouped by 3D genomic feature to generate pooled datasets. Then, we performed correlation analyses as previously described (39, 53). Briefly, the genome was partitioned into equal bins of defined size, within which the proportion of sequence covered by each feature of interest was determined. The global pairwise correlations between feature densities were performed to generate Spearman correlation coefficients and p values. These analyses were carried out using a breadth of bin sizes (20 kb, 50 kb, and 100 kb) to account for the differences among individual 3D genomic features in terms of their number of regions, median region size, and proportion of genome covered (Table S1).

We assembled 18 domain datasets from six studies (5, 6, 83, 85, 86, 93) covering 14 human cell types, including human embryonic and induced pluripotent stem cells as well as fetal cells, differentiated and cancer cell lines representing different germ layers (Table S1). Individually, these domain datasets cover varying percentages of the genome, ranging from 40.07% (HMEC) to 87.72% (SK-N-SH). Pooling the datasets together and collapsing the overlapping intervals resulted in a single dataset (hereafter named “pooled domains”) spanning 98.06% of the genome (Table S1). Pooled domains show predominantly significant, positive correlation with genes of most evolutionary eras, UCEs, and HARs (6.27×10^−35^ ≤ p ≤ 0.022), except mammal genes (p = 0.654) (Fig. 1F and Table S2). When examined by individual cell types, ancient genes and UCEs continue to display mainly significant, positive correlations with domains (0 ≤ p ≤ 0.746; Fig. S2 and Table S2). This observation is aligned with previous findings that UCEs and clusters of highly conserved noncoding elements, which may have regulatory function, coincide with domains (39, 40). Notably, annotated Ensembl ENSP genes also closely match the trend in correlations between ancient genes and domains (0 ≤ p ≤ 0.078; Fig. S2 and Table S2). Interestingly, less conserved era genes (metazoan, chordate, mammal, and primate) and fast-evolving HARs have more variable strengths of correlations with domains across cell types (2.34×10^−92^ ≤ p ≤ 0.988; Fig. S2 and Table S2). Taken together, these observations suggest that conserved elements have more consistent relationships with domains, while less conserved elements show more variable relationships with domains across cell types.

We continued our analyses using 36 datasets of loop anchors from various studies (6, 19, 83, 84, 87–92). These datasets span 36 distinct cell types ranging from H1 human embryonic stem (ES) cells, ES-cell-derived lineages, fetal cells, to differentiated and cancer cell lines (Table S1). Loop anchors cover a smaller portion of the genome than domains; these individual datasets range from 1.97% (RPE1) to 9.83% (aorta). Again, we pooled the loop anchor datasets together and collapsed the overlapping intervals to generate a dataset named “pooled loop anchors,” which covers 56.55% of the genome (Table S1). Similarly to pooled domains, pooled loop anchors display mostly significant, positive correlations with genes of most eras, UCEs, and HARs (1.08×10^−172^ ≤ p ≤ 0.003), except for mammal genes, whose correlation is significantly negative (p = 6.03×10^−5^) (Fig. 1F and Table S2). Notably, HARs have previously shown enrichment in loops specific to human compared to rhesus macaque and mouse (94). While the observed significant, positive correlation in UCEs differs from a former study (39), which displayed a non-significant relationship between UCEs and loop anchors, this result can be explained by the increase in the number of datasets used in this analysis (36 datasets here compared to previously used eight datasets). Our observation that UCEs are positively correlated with pooled loop anchors is consistent with the regulatory activity of some UCEs (95–103) and with loops facilitating enhancer-promoter contacts (6). When separated by cell type, UCEs together with HARs and less conserved era genes (metazoan, chordate, mammal, primate) have variable relationships with loop anchors (8.65×10^−118^ ≤ p ≤ 0.965; Fig. S3 and Table S2). On the other hand, ancient and ENSP genes show a robust, significant positive correlation with loop anchors across most cell types (0 ≤ p ≤ 0.944; Fig. S3 and Table S2). In addition, we noted that relationships of genes of different eras and HARs with loop anchors remain persistent across some cell types, mostly corresponding to Hi-C datasets produced with the same methodology (Fig. S3 and Tables S1 and S2). Overall, ancient genes have the most consistent relationships, while younger genes, HARs, and UCEs have more variable relationships with loop anchors across cell types.

Finally, we turned to boundary datasets from multiple studies (5, 84, 88, 92). These boundary datasets cover 20 cell types and primary tissues (Table S1). Individual datasets cover portions of the genome more comparable to loop anchors than domains ranging from 2.61% (aorta) to 3.95% (hESC). A dataset of pooled boundaries covers 22.5% of the genome (Table S1). This pooled dataset is significantly positively correlated with genes of different eras (2.62×10^−136^ ≤ p ≤ 2.90×10^−9^), consistent with transcription start sites being enriched at boundaries (5). Conversely, pooled boundaries are significantly negatively correlated with control Igen ORFs (p = 9.06×10^−18^) and Igen Non-ORFs (p = 2.11×10^−14^), UCEs (p = 1.81×10^−6^), and HARs (p = 1.76×10^−25^) (Fig. 1F and Table S2). These observations in UCEs and HARs are different from those found with pooled domains and loop anchors (Fig. 1F) and are consistent with the depletion of UCEs at boundaries (39). Furthermore, in distinct contrast to domains and loop anchors, genes of different eras, UCEs, and HARs all show remarkable consistency in their correlations with boundaries across cell types (Fig. S4 and Table S2). Strikingly, genes display predominantly significant, positive correlations with boundaries (2.19×10^−110^ ≤ p ≤ 0.306), as opposed to HARs with entirely significant, negative correlations (8.91×10^−12^ ≤ p ≤ 0.009) and UCEs with significant or non-significant negative correlations (6.24×10^−4^ ≤ p ≤ 0.136) (Fig. S4 and Table S2).

Additional correlation analyses with different bin sizes (20 and 100 kb; Tables S3 and S4) provide similar results to 50-kb results (Table S2) for boundaries as well as domains and loop anchors. Moreover, the control sets of Igen ORFs and Igen Non-ORFs, with no evidence of regulated transcription, support these findings with era genes by displaying similar correlations regardless of the inspected 3D genomic feature or cell type (Fig. 1 and Figs. S2–S4 and Table S2). Taken together, the relationships between genomic regions of varying sequence conservation and boundaries are consistent yet distinct, while the relationships with domains and loop anchors are variable across cell types. This variability, more prominent with evolutionarily younger genes and fast-evolving HARs, may suggest their potential role in tissue-specific programs.

### Gene expression changes with evolutionary age

**Fig. 2.**
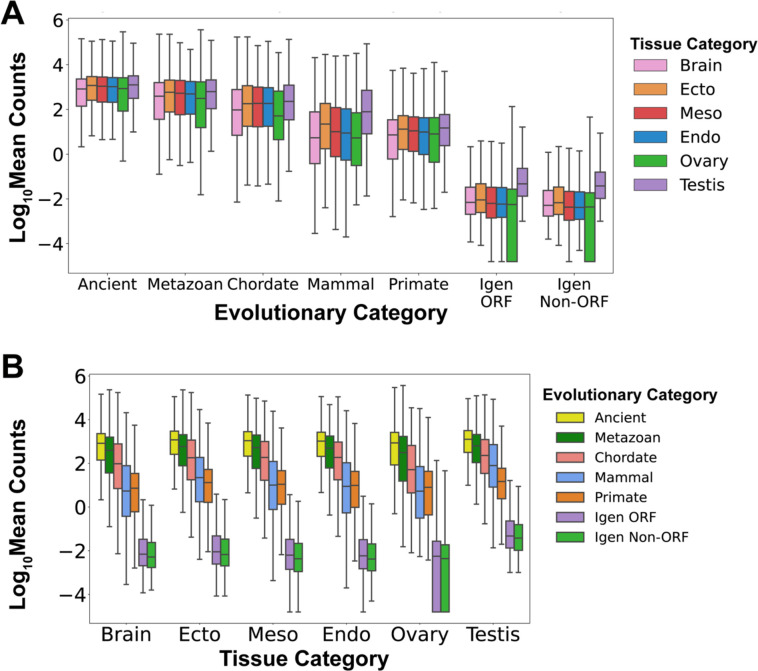
Transcriptional levels vary with gene age and tissue of origin. The expression of human protein-coding genes is measured as the log10 of the mean counts of RNA transcripts across 54 human tissues from the GTEx database (104). (*A*) RNA expression levels vary as a function of evolutionary category, increasing from youngest to oldest genes, and higher in all genes than in control non-genic sequences (Igen ORF, Igen Non-ORF). Testis expression is higher than expression in other tissues in control non-genic sequences. (*B*) RNA expression levels in 6 tissue categories: brain, 3 germ layers (ectoderm, mesoderm, endoderm), and 2 germline tissues (ovary, testis). Within every tissue category, expression increases from youngest genes to oldest genes and is higher in genes than in control non-genic sequences.

Due to the interplay between the 3D organization of the genome and gene expression, we measured the transcription of genes and intergenic control sequences using the Genotype to Tissue Expression (GTEx) database, which contains RNA sequencing data from 54 human adult tissues (Table S5) (104). We grouped the data into six categories: three germ layers (ectoderm, mesoderm and endoderm), germ line (ovary, testis), and the brain, since some evolutionarily novel genes function in neurons (59, 65, 70, 105). First, we examined the RNA expression of genes as a function of their evolutionary age (Fig. 2A Mean Counts, and Fig. S5A transcripts per million (TPM)). We found that gene expression changes with evolutionary era, such that, in all tissue categories, ancient genes are expressed at the highest levels while primate genes are expressed at significantly lower levels (Tables S5 and S6; for example, Ectoderm: Ancient 2709 ± 56, Primate 118 ± 117, average ± standard deviation, p < 2.225×10^−308^). Moreover, the expression of all genes is 100–1000 times higher than that of control Igen ORFs and Igen Non-ORFs, which reflect the transcriptional background of the genome (Tables S5 and S6; for example, Ectoderm: Primate 118 ± 117, Igen Non-ORF 0.23 ± 0.04, average ± standard deviation, p < 2.225×10^−308^).

Second, we examined the RNA expression of genes within 6 tissue categories (Fig. 2B and Fig. S5 B and C) and found that within each category there is a similar evolutionary progression of gene expression, such that ancient genes are expressed at higher levels than evolutionarily novel genes. Interestingly, within the testis, the expression of genes is higher than in other tissues, as observed in other species such as fruit fly (66). We observed that, for most genes of all eras, the expression of genes in the brain is similar or lower than in other tissues (Fig. S5 B and C). Within tissue categories, gene expression can vary substantially between individual tissues (Fig. S6). Within the brain, cerebellar hemispheres express the highest RNA levels of ancient genes while the amygdala and *substantia nigra* express the lowest RNA levels of ancient genes (Fig. S6). Additionally, we observed that, while the expression of genes generally increases with gene length (short genes are expressed at lower levels than long genes), within each gene length decile the evolutionary progression from ancient genes expressed at high levels to young genes expressed at low levels is mostly maintained (Fig. S5D). Taken together, these data show that gene expression increases with evolutionary age and that all protein-coding genes are expressed at levels much higher than the transcriptional background of the genome.

### Era genes are primarily associated with prominent cell type-specific processes

Given the distinct relationships between regions of varying sequence conservation and 3D genome structure across cell types, we next investigated the functional associations of those regions. Previous work by Domazet-Lošo and Tautz associated Ensembl annotated genes grouped by their evolutionary age with specific gene ontology (GO) terms (106). Due to the complexity of our datasets, which comprise recently curated annotated genes and as yet unannotated genes, we used the Genomic Regions Enrichment of Annotations Tool (GREAT) (107) to assess GO term enrichments in genomic coordinates of interest. This approach allowed inspection of different regions including HARs, UCEs, and unannotated genes, particularly primate genes, without a need to rely on gene identifiers. We observed that metazoan genes are enriched for GO terms related to signaling pathways against all era genes as a background (Fig. S7A). Meanwhile, chordate genes are associated with immune response-related processes (Fig. S7B). As for mammal and primate genes, both are linked to keratinization and keratinocyte differentiation processes (Fig. S7 C and D). However, mammal genes are also associated with defense response to other organisms (Fig. S7C). Ancient genes do not show enrichment in GO terms, most likely since they form a majority (69.25%) of all era genes. Together, these observations are consistent with a previous report (106) and suggest that various categories of era genes are predominantly associated with distinct cell type-specific GO terms.

### Evolutionarily older regions show stable functional associations within 3D genomic features

**Fig. 3.**
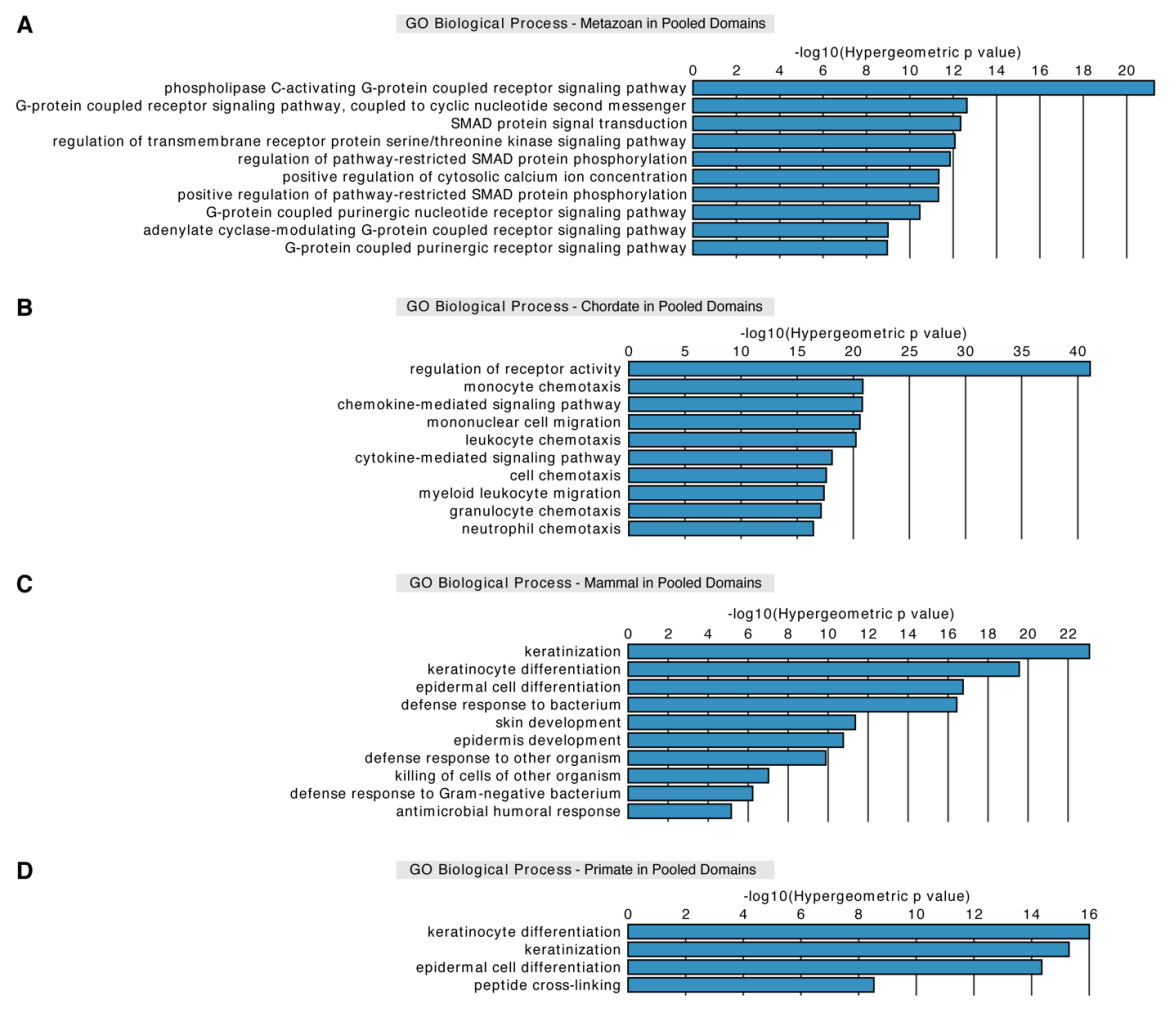
Genes of different evolutionary eras are associated with prominent cell type-specific processes in pooled domains. GREAT analysis for different era genes that overlap pooled domains with GO terms associated with (*A*) signaling pathways for metazoan genes, (*B*) immune response-related processes for chordate genes, (*C*) skin development and defense response to other organisms for mammal genes, and (*D*) keratinocyte differentiation for primate genes. (*A-D*) Analysis performed against all era genes as a background.

Having the corresponding functional associations of era genes, we examined whether these associations are related to positioning of era genes within 3D genomic features. Unsurprisingly, era genes in pooled domains are associated with the same GO terms (Fig. 3 A–D) as with the entire genome (Fig. S7). Pooling the domains across cell types may include regions that are domains in certain cell types but are boundaries and loop anchors in other cell types, and therefore incorporates more than 99% of the genes. In pooled loop anchors, metazoan and chordate genes still maintain very similar enrichment of GO terms (Fig. S8 A and B) as noted with genes in pooled domains (Fig. 3 A and B). On the other hand, evolutionarily younger mammal and primate genes lose association with defense response to other organisms and peptide cross-linking, respectively (Fig. S8 C and D). As for pooled boundaries, we observed a notable shift in the gene eras that exhibit GO term enrichment. For instance, ancient genes in pooled boundaries appear mainly associated with protein localization to telomeres as well as various ncRNA processes (Fig. S9A), consistent with the enrichment of housekeeping genes at boundaries (5). Meanwhile, metazoan and chordate genes in pooled boundaries show stable association with signaling pathways and immune response-related processes, respectively (Fig. S9 B and C). Interestingly, evolutionarily recent mammal and primate genes in pooled boundaries do not display any GO term enrichments. Overall, the functional associations of older metazoan and chordate genes seem to be stabilized and not affected by their positioning within 3D genomic features. In contrast, functional associations of younger mammal and primate genes may change depending on 3D genomic feature these genes overlap, suggesting sensitivity to their positioning within genome architecture.

We next compared our functional associations of era genes in various 3D genomic features with those of UCEs and HARs. Previous studies suggest that specific subsets of UCEs are associated with RNA processing and developmental processes (35, 39, 108–111). In line with this, we observed that all UCEs in pooled domains, boundaries, or loop anchors maintain consistent stable enrichment for GO terms related to transcription and RNA metabolic processes (Figs. S8E, S9D, and S10A) (35, 110, 112). HARs in pooled domains, which comprise all but one HAR in our dataset, are associated with neuronal processes, cartilage formation, and thymus development (Fig. S10B), consistent with functional implications of HARs due to their increased divergence in humans (42–44, 46–48, 50, 52). As for HARs in pooled loop anchors, associations are minorly altered to those with HARs in pooled domains (Fig. S8F). In pooled boundaries, HARs do not show enrichment for GO terms, which corresponds to the strong negative correlation between HARs and pooled boundaries (p = 1.76×10^−25^; Fig. 1D). Together, these results point to sensitivity in positioning of HARs within 3D genome architecture similar to evolutionarily recent mammal and primate genes.

### The functional associations of regions of varying sequence conservation are more sensitive to disease state in loop anchors than in domains

**Fig. 4.**
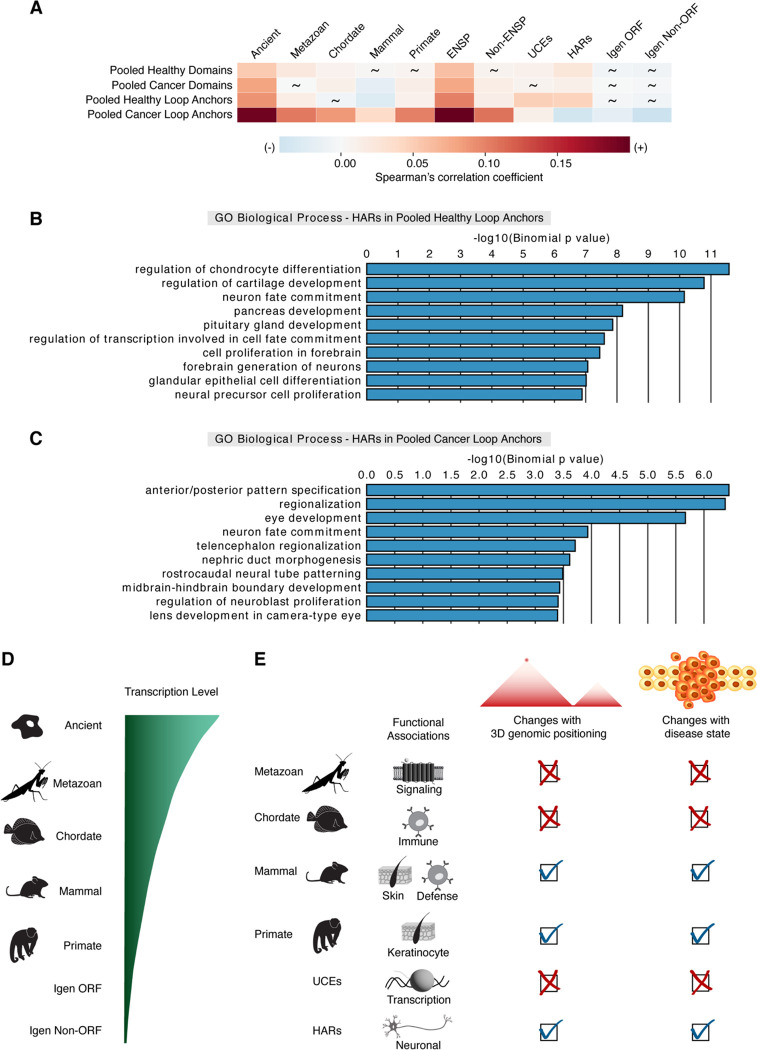
The relationship between genomic regions of different sequence conservation and 3D genome organization in healthy versus disease states. (*A*) Correlations of regions of varying sequence conservation with pooled domains and pooled loop anchors in healthy versus cancer states. Spearman correlation analysis was performed by partitioning the genome into 50-kb bins. Spearman correlation coefficients indicated by a heatmap. Non-significant p values are depicted by tildes. Control datasets include ENSP genes, Non-ENSP genes, Igen ORFs, and Igen Non-ORFs. (*B* and *C*) HARs that overlap pooled loop anchors in (*B*) healthy and (*C*) cancer states against whole genome background share associations with neuronal GO categories. However, such HARs in healthy state are enriched for GO terms associated with cartilage, pancreas, and gland development, while HARs in cancer are associated with pattern specification, eye, and kidney development. (*D*) Evolutionary progression of transcription levels: RNA expression increases with time from youngest genes (primate) to oldest genes (ancient) and is higher in genes than in control non-genic sequences (Igen ORF, Igen Non-ORF). (*E*) Schematic of the functional associations of genomic regions of varying sequence conservation and their changes with 3D genomic positioning and disease state. Change is indicated by a check mark and consistency is depicted by an X.

Due to potential changes in chromatin folding and gene regulation in disease states (17, 20, 113), we next asked whether healthy and diseased cancer states can impact the positioning and the functional associations of regions of varying sequence conservation within 3D genomic features. Correlations of pooled domains with metazoan, mammal, and primate genes as well as UCEs change from nonsignificant in one state to significant in the other (2.29×10^−7^ ≤ p ≤ 0.870; Fig. 4A and Table S2). Despite this variation in 3D positional relationships, the functional associations of those regions seem mainly unaffected. Broadly, era genes, UCEs, and HARs in both pooled healthy domains (Fig. S11) and pooled cancer domains (Fig. S12) are enriched for very similar GO terms, as seen in all pooled domains (Fig. 3 and Fig. S10). For pooled loop anchors, we observed changes in the correlations for chordate and mammal genes along with HARs between healthy and cancer states (Fig. 4A). As for functional associations, although GO terms associated with era genes, UCEs, and HARs in pooled healthy loop anchors (Fig. 4B and Fig. S13) closely resemble those found with all pooled loop anchors (Fig. S8), GO term enrichments in pooled cancer loop anchors can show notable shifts depending on the class of region (Fig. S14). For instance, older metazoan and chordate genes and UCEs in pooled loop anchors have largely similar GO term enrichments in cancer (Fig. S14 B, C, and D) and healthy (Fig. S13 A, B, and E) states, potentially suggesting the exploitation of signaling pathways by cancer cells (114). In contrast, younger mammal and primate genes in pooled cancer loop anchors show no GO term enrichments. As for HARs in pooled loop anchors, we noted changes in GO term enrichments between healthy (Fig. 4B) and cancer states (Fig. 4C). While both states are still associated with neuronal-related processes, the individual GO terms are often state-specific. These diverse associations of HARs are in line with their implications in human neurodevelopment and disease (115, 116). In summary, the functional associations of evolutionarily recent mammal and primate genes in pooled loop anchors seem to be lost in disease, suggesting that cancer cells may better exploit the stable functional associations of older genes.

## Discussion

Our study reveals that the relationships between genes of different evolutionary ages and their positioning within domains and loop anchors are variable, while the correlations of those era genes with boundaries display remarkable consistency across cell types. The transcription levels of genes also increase with increasing evolutionary age in all tissue categories (Fig. 4D). As for the functional associations of era genes, they are predominantly related to distinct cell type-specific processes. The functional associations of more recently evolved genes as well as HARs are sensitive to their 3D genomic positioning. This sensitivity is especially prominent in loop anchors compared to domains in disease state (Fig. 4E). Loop formation has been implicated in demarcating domains and facilitating contacts between enhancers and promoters (6, 9, 12). Although domain and loop structures may be changing, at least half of them may be preserved across cell types (5, 6, 117). Ancient genes exhibit more consistent positive correlations with both domains and loop anchors between cell types, while less conserved, younger genes, with their marked increase in correlation variability, may be linked to cell type-specific chromatin organization. Furthermore, evolutionarily younger genes are associated with cell type-specific functions suggesting their potential role in tissue-specific programs. Interestingly, previous studies have shown that many loops involving enhancers and promoters may be tissue-specific during differentiation, and therefore related to cell type-specific gene regulation (118, 119). Hence, the relationships of these recent era genes with 3D genomic features may be implicated in evolution of tissue-specific genome activity. Moreover, modifications to the dynamic 3D regulatory landscape may even shape species-specific programs (120–125).

Notably, measuring transcriptional output with GTEx shows an evolutionary progression of gene expression: young genes are expressed at lower levels than ancient genes, yet at much higher levels that background genomic transcription. The output of gene regulation by way of using various gene regulatory elements, including 3D genome organization elements, is gene expression. Thus, the placement of 3D genome organization elements may be used to control gene expression at the RNA level, as transcriptional complexes must access DNA to produce RNA. Interestingly, for genes of all ages in the six tissue categories we considered, RNA levels are highest in testis, a tissue where DNA is loosely packed with protamines instead of histones (126), and are unexpectedly lowest in the brain, where gene expression is tightly regulated but there are many cell types. Indeed, some brain areas such as the cerebellum do have high RNA expression. The similar evolutionary progression of RNA levels in all tissues suggests RNA levels are regulated by the 3D genome organization elements that may be implicated in opening chromatin, and thus in part independently of transcription factors – while a few transcription factors, like CTCF (22), change genome organization, most transcription factors regulate expression differently, by cooperatively aiding the assembly of the RNA polymerase II transcriptional complex. These two facets of gene regulation support transcription of a new gene, and are stabilized evolutionarily if the gene finds a function and perdures evolutionarily. Additionally, longer genes of all ages are expressed at higher levels than shorter genes of all ages, suggesting gene size, usually higher in neuronal genes (80, 127), is associated to gene expression level independently of gene age.

The functional associations found with genes of each era are intriguing in light of the biological processes that arose at different times in evolution (78, 106). In line with this notion, the SMAD-dependent and G-protein coupled receptor signaling pathways associated with metazoan genes are often involved in altering gene expression related to tissue differentiation, which is critical for development of multicellular animals (128). Chordate genes as well as mammal genes link to several immune processes which may mediate defense responses against pathogens (129, 130). Both mammal and primate genes are associated with keratin processes, possibly related to hair formation which may be integral to heat retention and homeothermy (131). Interestingly, these links to immune response and skin development are further supported by the functional associations of fast-evolving genes and regulatory landscape in mammals facilitating an organism’s interactions with the environment (51, 132). Overall, the emergence of genes at different evolutionary times may be linked to novel biological processes gained at different levels of the phylogenetic hierarchy (78, 106).

Disruptions of the 3D genome architecture can play roles in various diseases, especially when interactions between regulatory regions are affected (13, 14, 17, 20, 119). In particular, the identity and evolutionary age of the genes impacted by such alterations may be integral to understanding a disease. For instance, previous reports indicate a significant over-representation of disease-associated genes, including cancer-associated genes, that predate or originate with the emergence of Metazoa (106, 114). Interestingly, we observed little change in the functions associated with metazoan genes in loop anchors between healthy and cancer states, possibly related to the exploitation of signaling pathways by cancer cells in multicellular organisms (114). As for evolutionarily recent mammal and primate genes in loop anchors, both lose all functional GO term enrichments in cancer, consistent with the under-representation of such genes among cancer and other disease-associated genes (106, 114). Nevertheless, fast-evolving HARs in loop anchors maintain functional associations with neuronal processes in both healthy and cancer states, reminiscent of the other disease implications of HARs, including in neurological disease (115, 116).

In conclusion, we describe the functional significance of the 3D genomic positioning of genes of different evolutionary ages. The evolution of genes and their regulatory regions within the 3D genome may present different lenses through which the determinants of yet unresolved genetic diseases may be elucidated.

## Materials and Methods

### Human sequence datasets

As in other studies (80, 133), we retrieved 19,334 human annotated proteins from the Ensembl 89 database (www.ensembl.org), keeping the oldest and longest isoform per protein-coding gene. Additionally, we used 2,102 human unannotated protein sequences that are proteogenomically confirmed (74–77). Furthermore, we used two kinds of control sequences. The first are Igen ORFs, localized in the non-genic non-repetitive part of the human genome, starting with a methionine (Met) codon and having at least 40 non-STOP codons, followed by a STOP codon. These sequences have no known evidence of transcription or translation but could in principle encode proteins, based on their DNA sequence. The second type of controls are intergenic DNA sequences that are random sequences from the non-repetitive part of the human genome that do not overlap with any selected ORFs or known protein-coding genes, do not start with Met, may have STOP codons and are not expected to encode open reading frames (Igen Non-ORFs). Igen Non-ORFs were chosen to have the same DNA sequence length distribution as Igen ORFs.

### Gene age estimation

We estimated the evolutionary age of protein-coding genes by phylostratigraphy (78, 79), which classifies genes based on their taxonomic restriction (80). For genes encoding proteins of minimum 40 amino acids, the protein sequence was used to query the NCBI NR database (February 2017) and find the most taxonomically distant species in which a sufficiently similar protein sequence exists, such that the e-value of that most distant BLASTP hit is less that 10^−3^ (80). Sequence similarity was evaluated with BLASTP (134) and HMMER (135). Minimal gene age was assessed as the time when the most recent common ancestor of the query species and the most distant species lived. Within the human lineage, there are 31 taxonomical nodes, corresponding to 31 phylostrata (PS; NCBI Taxonomy, https://www.ncbi.nlm.nih.gov/Taxonomy/Browser/wwwtax.cgi?id=9606), and their timing was estimated using TimeTree (136). The 31 phylostrata were grouped into 5 large-scale evolutionary eras: Ancient (PS 1–3), Metazoan (PS 4–7), Chordate (PS 8–17), Mammal (PS 18–22), Primate (PS 23–31). Human genes also present in bacteria are in PS 1 and belong to the Ancient era, while human genes present only in humans are in PS 31 and thus belong to the Primate era. For each gene, only the oldest and longest protein isoform was kept.

### UCE and HAR datasets

The UCE dataset includes 896 elements from comparisons between human-mouse-rat (HMR), human-dog-mouse (HDM), or human-chicken (HC) genomes as previously reported (39, 53, 81). The genomic coordinates for HARs were obtained from Girskis *et al.* (43) and converted to the hg19 genome assembly using the UCSC LiftOver tool (https://genome.ucsc.edu/cgi-bin/hgLiftOver). Any overlapping intervals were collapsed to avoid including the same region more than once.

### Hi-C datasets

The coordinates for Hi-C annotated genomic features, including domains, boundaries, and loop anchors, were retrieved from published datasets as indicated in Table S1 (5, 6, 19, 83–93). The genomic coordinates were processed as previously described (39). Namely, when applicable, the Hi-C genomic annotations were lifted over to the hg19 genome assembly using LiftOver. To ensure that any given region was counted only once, overlapping intervals were collapsed to generate final pooled and individual datasets for domains, boundaries, and loop anchors that may differ from previously published original ones. Information about these final datasets, including number of regions, median region size (bp), coverage (bp), and proportion of genome covered (%), is provided in Table S1. Eres *et al.* (85) provided four iPSC datasets derived from four individuals, while Dixon *et al.* (5) and Rao *et al.* (6) each provided one dataset of IMR90 using dilution and in situ Hi-C protocols, respectively. Rubin *et al.* (90) provided three Hi-C datasets corresponding to different stages of keratinocyte differentiation (days 0, 3, and 6). Dixon *et al.* 2012 (5) and Dixon *et al.* 2015 (84) each provided a Hi-C dataset for H1 human embryonic stem cells. Due to the methodological variations among these studies such as Hi-C protocol, quantity of input material, and sequencing depth, we examined each dataset individually, grouped by a Hi-C feature (e.g. Pooled Domains), and grouped additionally based on healthy or disease state (e.g. Pooled Cancer Domains).

### Correlation analyses between 3D genomic features and regions of varying sequence conservation

Correlation analyses were performed using a previously established method (39, 53). Briefly, the genome was first split into bins of fixed size. In each bin, the proportion of sequence covered by a 3D genomic feature or regions of varying sequence conservation was calculated. Then, the global correlations among feature densities of interest were determined. The Spearman correlation coefficients and the corresponding p values were reported for a pairwise comparison between two features (e.g. between era genes, UCEs, or HARs and a 3D genomic feature) using three different bin sizes (20 kb, 50 kb, 100 kb; Tables S2, S3, and S4). The Spearman correlation coefficients were also indicated by a heatmap.

### GTEx analysis

To determine the level of RNA expression of all human protein-coding genes encoding proteins of at least 40 amino acids and of control intergenic sequences, a RNA-Seq calling pipeline was used to calculate the number of reads per gene per sample broken down by tissue. GTF files with all sequences (annotated and unannotated Genes, Igen controls) were run through the GTEx RNA-Seq calling pipeline of the Broad Institute (https://github.com/broadinstitute/gtex-pipeline). The control intergenic sequences inform on the background transcription of the human genome. Deseq2 normalization of means was used (https://github.com/broadinstitute/pyqtl) to account for sample-level sequencing depth between individuals and RNA composition. From this, we obtained a mean read count value for each sequence for each of the 54 tissues of GTEx. These tissues were aggregated into the following tissue categories: three germ layers (ectoderm, mesoderm, endoderm), two germline tissues (ovary, testis) and one tissue of special interest to the authors (brain). To avoid overweighting brain regions in ectoderm, only cortex and cerebellum were included (and these tissues were also included in brain, along with 11 other brain regions). Similar analyses were conducted with transcripts per million (TPMs) per gene, as available from the online resource of GTEx for annotated genes alone (v8, median TPM counts per gene). TPM data was downloaded directly from the GTEx v8 Browser’s download page (https://storage.googleapis.com/adult-gtex/bulk-gex/v8/rna-seq/GTEx_Analysis_2017-06-05_v8_RNASeQCv1.1.9_gene_median_tpm.gct.gz), with median TPM counts per 54 tissues per Ensembl annotated gene. The purpose of the TPM analysis was checking if the changes with evolutionary age observed with Mean Counts remain the same with TPMs, which better accounts for transcript length but does not work as well for small genes.

Transcripts of human genes encoding proteins of at least 40 amino acids (19,334 annotated protein-coding genes, 2,102 unannotated proteogenomically confirmed genes) and control sequences were counted in 54 human adult tissues (GTEx database) using GRCh38 genomic coordinates for all sequences. Of 20,000 randomly picked Igen ORFs (ten sets of 2,000 each), for our analysis we used two sets of 2,000 each (4,000 sequences total), from which 3,916 Igen ORFs passed GTEx filtering consisting of not duplicating or overlapping other Igen ORFs, and cleanly mapping to GRCh37. Of 20,000 randomly picked Igen Non-ORFs, for our analysis we used two sets of 2,000 each (4,000 sequences total), from which we used 3,889 Igen Non-ORFs that were filtered to not include duplicates, not overlap other control Igen ORFs, Igen Non-ORFs, or genes, and map cleanly to GRCh37. The same approach was applied to 20,000 GRCh37 Igen ORFs and 20,000 Igen Non-ORFs, resulting in 3,924 Igen ORFs and 3,892 Igen Non-ORFs, which cleanly mapped to GRCh38 and passed the filtering described previously. A small number of genes (less than 1.3% of all annotated genes) do not map fully between GRCh37 and GRCh38 (247 genes with the same age distribution as all genes; 179 ancient, 32 metazoan, 25 chordate, 8 mammal, 3 primate). The data were analyzed as a function of gene evolutionary age. Statistical comparisons between gene age categories (genes of different eras and the controls) and between tissue groups were done with the Mann-Whitney U test, with p values corrected for multiple comparisons with the Benjamini-Hochberg correction. P values of 0 indicate numerical values smaller than 2.225×10^−308^. Data visualization was done using the Python library, Seaborn 0.12.2 (https://github.com/mwaskom/seaborn/tree/v0.12). Boxes are from the 25^th^ to the 75^th^ percentile; medians are indicated by horizontal line; whiskers indicate 1.5 times the interquartile range, with outliers not shown (but included in analysis). For visualization purposes, we applied a log10 data transformation, and handled 0.00 values by adding the smallest non-zero value to every log10 argument.

### GREAT analysis

Genomic Regions Enrichment of Annotations Tool (GREAT, version 4.0.4) (107) analysis was performed to assess gene ontology (GO) terms associated with genes in each era, UCEs, or HARs. This approach of using genomic coordinates of interest allowed inspection of HARs, UCEs, and yet unannotated novel genes, especially in the category of primate genes, that may have otherwise gone amiss with more conventional approaches that rely on gene names or identifiers (107). Furthermore, subsets of regions of varying sequence conservation that overlap with 3D genomic feature of interest were determined using pybedtools wrapped bedtools intersect (137, 138). The argument u=True ensured that the same region was written only once if any overlaps were found with 3D genomic feature. Such regions that intersected a 3D genomic feature were assessed for their association with GO terms against the relevant background. For genes in each era, the total list of all era genes was used as a background. In the case of UCEs and HARs, the whole genome provided by GREAT was used as a background.

## Supplementary Material

Supplement 1

## Data Availability

The publicly available datasets were obtained using accession numbers GSE71831 (83), GSE71072 (86), GSE63525 (6), GSE77565 (93), the 3D Genome Browser (3dgenome.org), dbGaP (GTEx, phs000424.v8.p2), and publications as provided in the Table S1.
