# Supplementary material for: Functional associations of evolutionarily recent human genes exhibit sensitivity to the 3D genome landscape and disease
Source: bioRxiv. 2024 Nov 22:2024.03.17.585403. Originally published 2024 Mar 17. Preprint. [Version 2] doi: 10.1101/2024.03.17.585403 (PMC10980080; doi:10.1101/2024.03.17.585403)
Supplement: Supplement 1 [file NIHPP2024.03.17.585403v2-supplement-1.pdf]

bioRxiv preprint doi: https://doi.org/10.1101/2024.03.17.585403; this version posted November 22, 2024. The copyright holder for this preprint (which was not certified by peer review) is the author/funder, who has granted bioRxiv a license to display the preprint in perpetuity. It is made available under aCC-BY 4.0 International license.

Supplemental information

Functional associations of evolutionarily recent human genes exhibit sensitivity to the 3D genome landscape and disease

Katherine Fleck, Victor Luria, Nitanta Garag, Amir Karger, Trevor Hunter, Daniel Marten, William Phu, Kee-Myoung Nam, Nenad Sestan, Anne H. O'Donnell-Luria, and Jelena Erceg

bioRxiv preprint doi: https://doi.org/10.1101/2024.03.17.585403; this version posted November 22, 2024. The copyright holder for this preprint (which was not certified by peer review) is the author/funder, who has granted bioRxiv a license to display the preprint in perpetuity. It is made available under aCC-BY 4.0 International license.

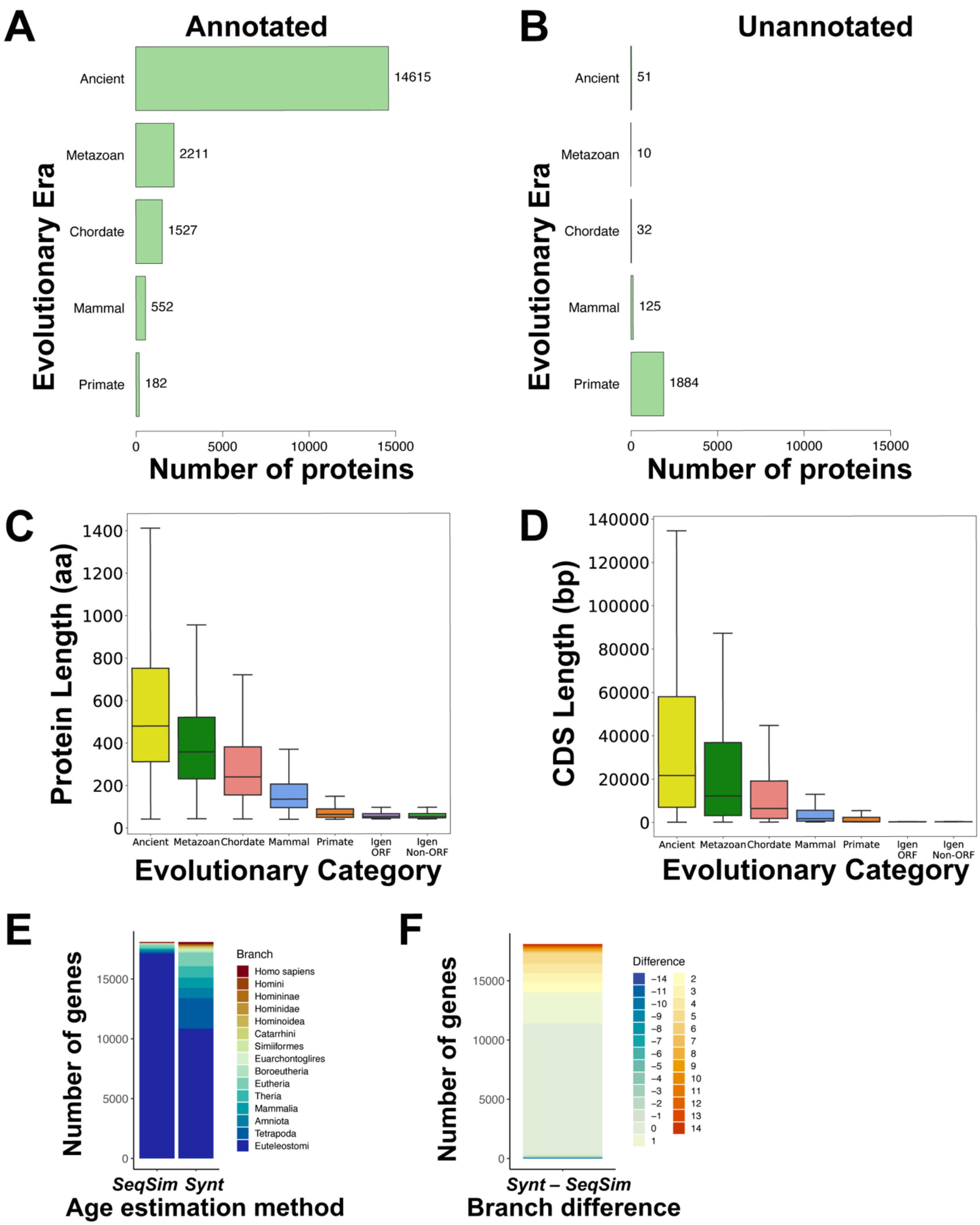

Figure S1. Gene numbers and lengths for each evolutionary era.

bioRxiv preprint doi: https://doi.org/10.1101/2024.03.17.585403; this version posted November 22, 2024. The copyright holder for this preprint (which was not certified by peer review) is the author/funder, who has granted bioRxiv a license to display the preprint in perpetuity. It is made available under aCC-BY 4.0 International license.

(A) Number of annotated human protein-coding genes in every evolutionary era (ancient, metazoan, chordate, mammal, primate). Most annotated genes are evolutionarily ancient. (B) Number of unannotated human protein-coding genes in every evolutionary era (ancient, metazoan, chordate, mammal, primate). Most unannotated genes are evolutionarily young. (C) Protein length is indicated in number of amino acids (AA). The evolutionarily youngest genes are much shorter than ancient genes and only slightly longer than control Igen ORF and Igen Non-ORF sequences. (D) The length of coding sequences (CDS) is indicated as the number of base pairs (bp). The evolutionarily youngest genes are shorter than ancient genes and similar to control Igen ORF and Igen Non-ORF sequences. (E-F) Comparison of gene age estimated by sequence similarity (SeqSim) and synteny (Synt) in 18,098 human genes. (E) The number of human protein-coding genes is indicated in 15 evolutionary branches used in the GenTree synteny-based database[1]. More genes appear evolutionary young by synteny than by sequence similarity. (F) Difference between the branch number of every gene as evaluated by synteny or sequence similarity. Most genes show no difference. A substantial fraction appears younger by synteny. Very few appear younger by sequence similarity.

bioRxiv preprint doi: https://doi.org/10.1101/2024.03.17.585403; this version posted November 22, 2024. The copyright holder for this preprint (which was not certified by peer review) is the author/funder, who has granted bioRxiv a license to display the preprint in perpetuity. It is made available under aCC-BY 4.0 International license.

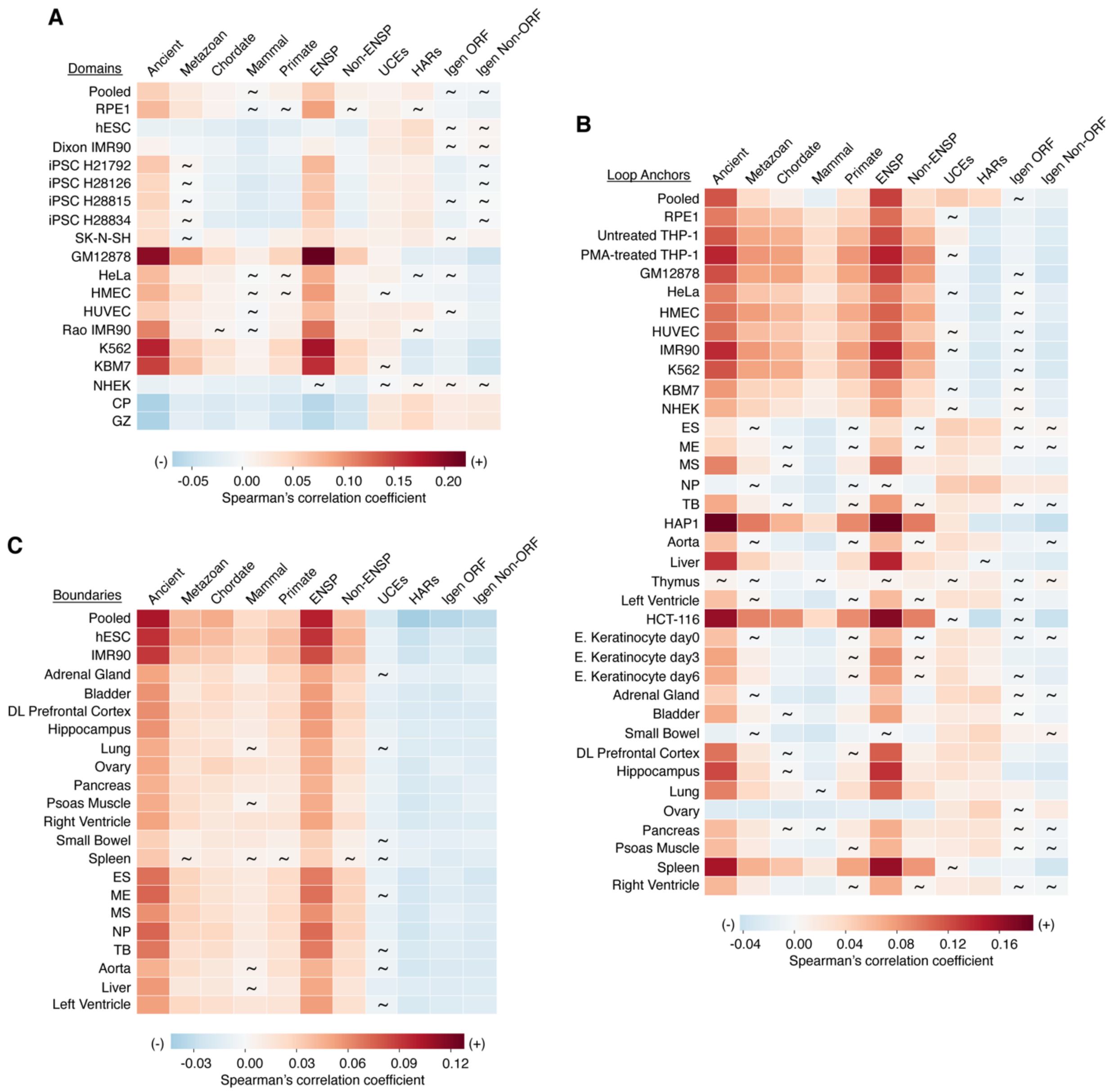

Figure S2. The relationships between 3D genomic features and various evolutionary genomic regions show stability or variation across cell types.

(A) Ancient genes, annotated genes, and UCEs are primarily significantly positively correlated with domains ($0 \leq p \leq 0.746$). Conversely, less conserved genes and fast-evolving HARs show variable relationships with domains across cell types ($2.34\times10^{-92} \leq p \leq 0.988$). (B) The relationships between loop anchors and regions of various sequence conservation show different

bioRxiv preprint doi: https://doi.org/10.1101/2024.03.17.585403; this version posted November 22, 2024. The copyright holder for this preprint (which was not certified by peer review) is the author/funder, who has granted bioRxiv a license to display the preprint in perpetuity. It is made available under aCC-BY 4.0 International license.

variation levels across cell types. Ancient and annotated genes have more consistent relationships with loop anchors by cell type ($0 \leq p \leq 0.944$), whereas less conserved genes, HARs, and UCEs have more variable relationships ($8.65\times10^{-118} \leq p \leq 0.965$). (C) The relationship of boundaries with era genes, UCEs, and HARs is highly consistent across cell types. Era genes are predominantly significantly positively correlated with boundaries ($2.19\times10^{-110} \leq p \leq 0.306$), while UCEs and HARs are non-significantly or significantly negatively correlated ($8.91\times10^{-12} \leq p \leq 0.136$). (A-C) Spearman correlation analysis was performed by partitioning the genome into 50-kb bins. Spearman correlation coefficients are indicated by a heatmap. Non-significant p values are depicted by tildes. Control datasets include annotated genes, unannotated genes, Igen ORFs, and Igen Non-ORFs. CP, cortical and subcortical plate; GZ, germinal zone; E., epidermal; DL, dorsolateral.

bioRxiv preprint doi: https://doi.org/10.1101/2024.03.17.585403; this version posted November 22, 2024. The copyright holder for this preprint (which was not certified by peer review) is the author/funder, who has granted bioRxiv a license to display the preprint in perpetuity. It is made available under aCC-BY 4.0 International license.

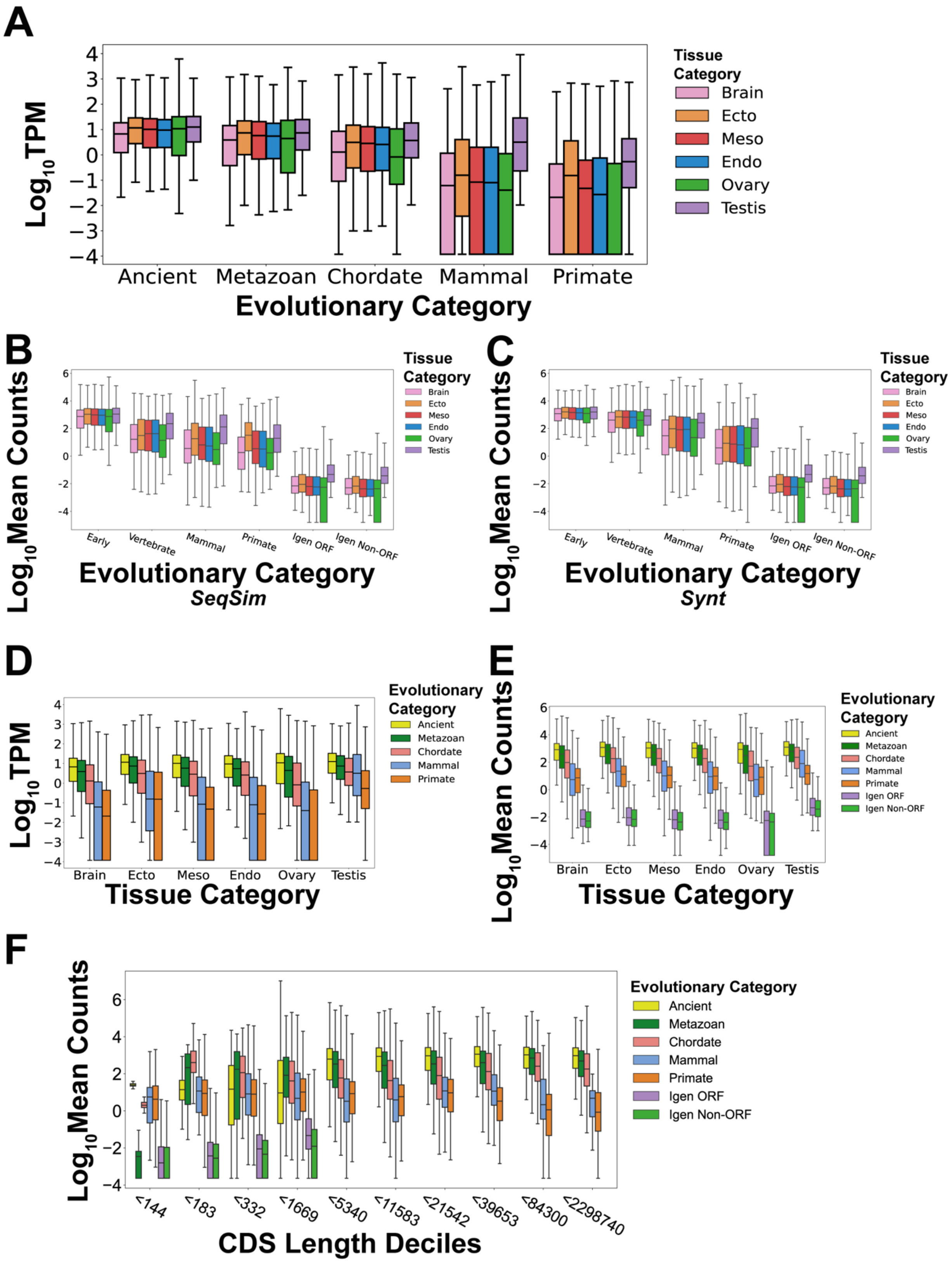

bioRxiv preprint doi: https://doi.org/10.1101/2024.03.17.585403; this version posted November 22, 2024. The copyright holder for this preprint (which was not certified by peer review) is the author/funder, who has granted bioRxiv a license to display the preprint in perpetuity. It is made available under aCC-BY 4.0 International license.

Figure S3. Transcriptional level variation with gene age, tissue of origin and gene length.

As in Figure 2, the expression of human protein-coding genes is measured for RNA transcripts across 54 human tissues from the GTEx database[2]. (A) RNA expression levels, measured as log10 of TPMs, increase from youngest to oldest genes (Table S3C). Within mammal and primate genes, expression in testis is higher than in other tissues. (B-C) RNA expression of human genes of different evolutionary ages as a function of the gene age estimation method – sequence similarity, SeqSim (B), synteny, Synt (C). Genes of different lineage restriction levels, or gene ages, are grouped into 4 eras (early, vertebrate, mammal, primate) that accommodate the 15 evolutionary branches used by GenTree[1]. Data are normalized counts of RNAs across 54 human tissues from the GTEx database[2]. The evolutionary change from low levels in young genes to high levels in early genes is very similar between the two methods. (D) RNA expression levels, measured as log10 of TPMs, in 6 tissue categories increase from youngest genes to oldest genes. (E) RNA expression levels in 6 tissue categories: brain, 3 germ layers (ectoderm, mesoderm, endoderm), and 2 germline tissues (ovary, testis). Within every tissue category, expression increases from youngest genes to oldest genes and is higher in genes than in control non-genic sequences. (F) RNA expression levels, measured as log10 of mean counts, increase with the gene length, shown as CDS length. Within 7 CDS length deciles, ancient genes are expressed at highest levels while primate genes are expressed at lowest levels. Within 3 of the shorter CDS length deciles, the highest expression is that of metazoan genes or chordate genes. Only the first 4 deciles have control non-genic sequences, and their expression is 100-1000 times lower than that of genes.

bioRxiv preprint doi: https://doi.org/10.1101/2024.03.17.585403; this version posted November 22, 2024. The copyright holder for this preprint (which was not certified by peer review) is the author/funder, who has granted bioRxiv a license to display the preprint in perpetuity. It is made available under aCC-BY 4.0 International license.

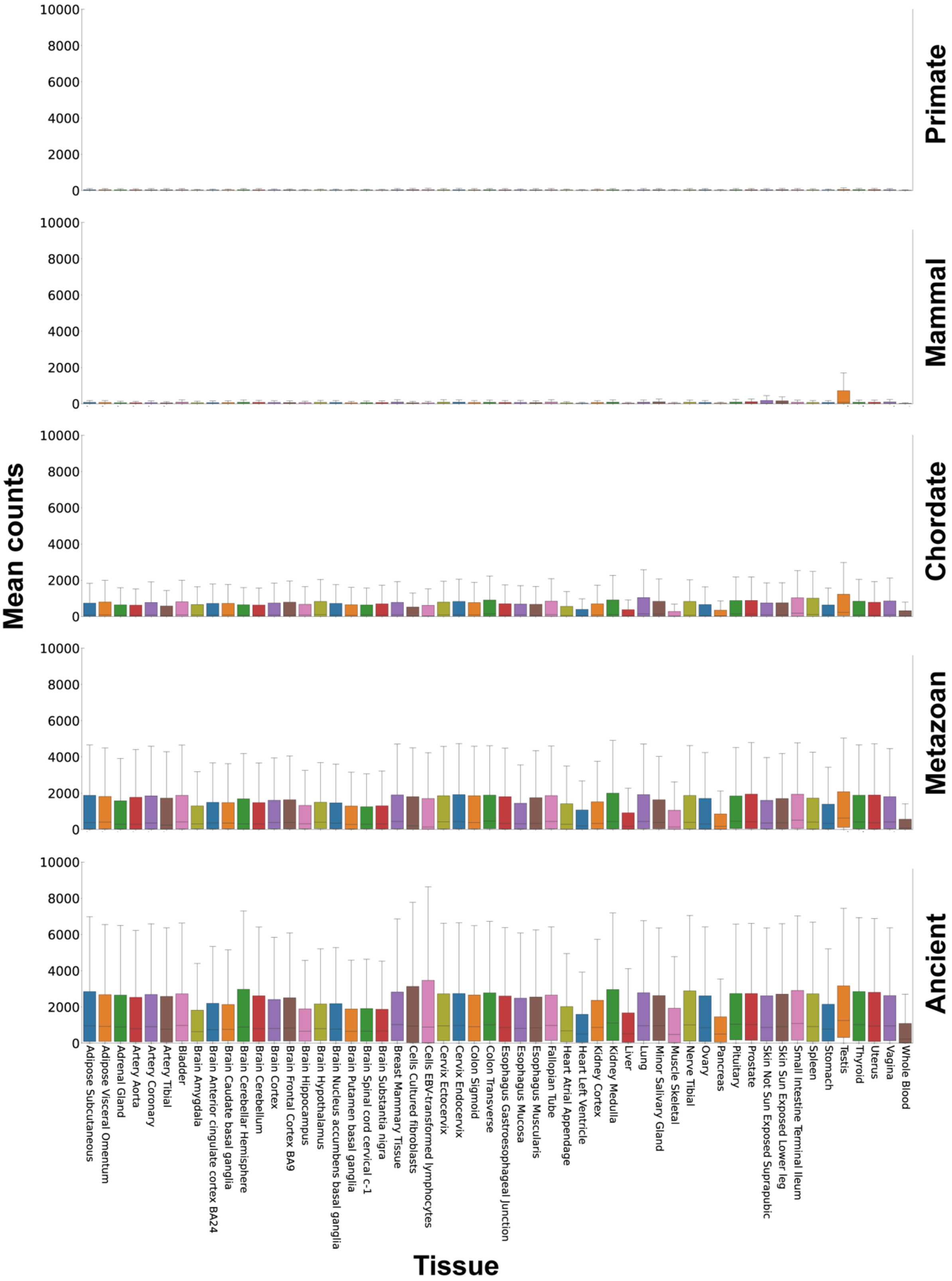

bioRxiv preprint doi: https://doi.org/10.1101/2024.03.17.585403; this version posted November 22, 2024. The copyright holder for this preprint (which was not certified by peer review) is the author/funder, who has granted bioRxiv a license to display the preprint in perpetuity. It is made available under aCC-BY 4.0 International license.

Figure S4. Gene expression in 54 human tissues and evolutionary eras.

Gene expression levels are measured as mean counts of RNA transcripts across 54 human tissues in 5 evolutionary eras, as in Figures 2 and S3. Gene expression increases evolutionarily from primate to ancient genes. With each evolutionary era, testis expression is typically highest, followed by other tissues which differ between eras.

bioRxiv preprint doi: https://doi.org/10.1101/2024.03.17.585403; this version posted November 22, 2024. The copyright holder for this preprint (which was not certified by peer review) is the author/funder, who has granted bioRxiv a license to display the preprint in perpetuity. It is made available under aCC-BY 4.0 International license.

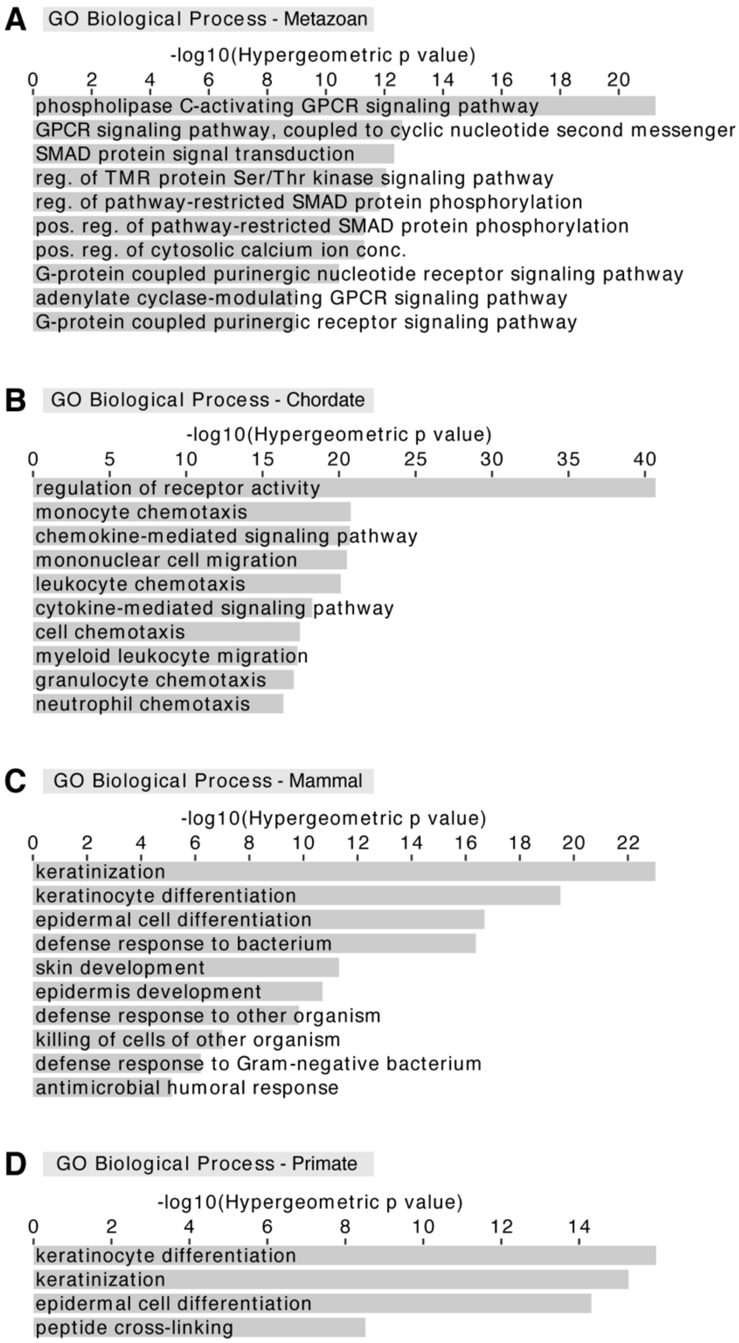

Figure S5. Functional associations of era genes.

GREAT analysis for (A) metazoan genes, (B) chordate genes, (C) mammal genes, and (D) primate genes. Those GO terms are associated with very similar processes as for era genes in pooled domains (Figure 3). (A-D) Analysis performed against all era genes as a background. Abbreviated

bioRxiv preprint doi: https://doi.org/10.1101/2024.03.17.585403; this version posted November 22, 2024. The copyright holder for this preprint (which was not certified by peer review) is the author/funder, who has granted bioRxiv a license to display the preprint in perpetuity. It is made available under aCC-BY 4.0 International license.

GO terms: GPCR, G-protein coupled receptor; reg., regulation; TMR, transmembrane receptor; Ser, serine; Thr, threonine; pos., positive; conc., concentration.

bioRxiv preprint doi: https://doi.org/10.1101/2024.03.17.585403; this version posted November 22, 2024. The copyright holder for this preprint (which was not certified by peer review) is the author/funder, who has granted bioRxiv a license to display the preprint in perpetuity. It is made available under aCC-BY 4.0 International license.

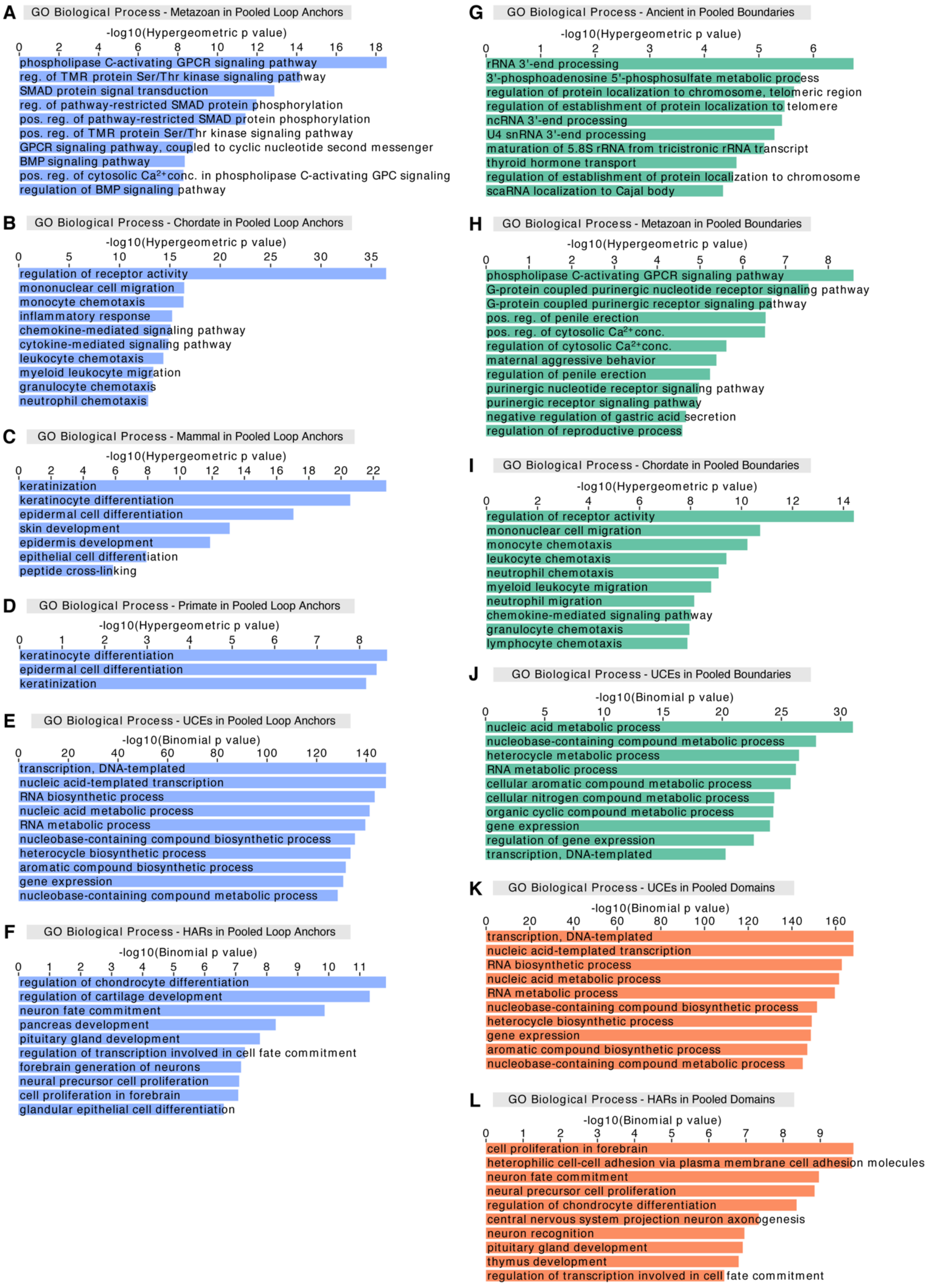

bioRxiv preprint doi: https://doi.org/10.1101/2024.03.17.585403; this version posted November 22, 2024. The copyright holder for this preprint (which was not certified by peer review) is the author/funder, who has granted bioRxiv a license to display the preprint in perpetuity. It is made available under aCC-BY 4.0 International license.

Figure S6. Functional associations of era genes, UCEs, and HARs within 3D genomic features.

Regions of varying sequence conservation overlapping pooled loop anchors are linked to GO terms associated with (A) signaling pathways for metazoan genes, (B) immune response-related processes for chordate genes, (C) skin development for mammal genes (note absence of defense response compared to Figure 3C), (D) keratinocyte differentiation for primate genes (note absence of peptide cross-linking compared to Figure 3D), (E) transcription and RNA metabolic processes for UCEs, and (F) neuronal processes, cartilage formation, pancreas development, and glandular epithelial cell differentiation for HARs. In pooled boundaries, enrichments for GO terms are related to (G) protein localization to telomeres and ncRNA processes for ancient genes, (H) signaling pathways and regulation of reproductive processes for metazoan genes, (I) immune response-related processes for chordate genes, and (J) transcription and RNA metabolic processes for UCEs. (K) UCEs in pooled domains are associated with transcription and nucleic acid metabolism, and (L) HARs in pooled domains are associated with neuronal processes, cartilage formation, and thymus development. Backgrounds: all era genes for individual eras of genes; whole genome for UCEs and HARs. Abbreviated GO terms: GPCR, G-protein coupled receptor; reg., regulation; TMR, transmembrane receptor; Ser, serine; Thr, threonine; pos., positive; conc., concentration; tricistronic rRNA transcript, tricistronic rRNA transcript (SSU-rRNA, 5.8S rRNA, LSU-rRNA). Blue, pooled loop anchors; green, pooled boundaries; orange, pooled domains.

bioRxiv preprint doi: https://doi.org/10.1101/2024.03.17.585403; this version posted November 22, 2024. The copyright holder for this preprint (which was not certified by peer review) is the author/funder, who has granted bioRxiv a license to display the preprint in perpetuity. It is made available under aCC-BY 4.0 International license.

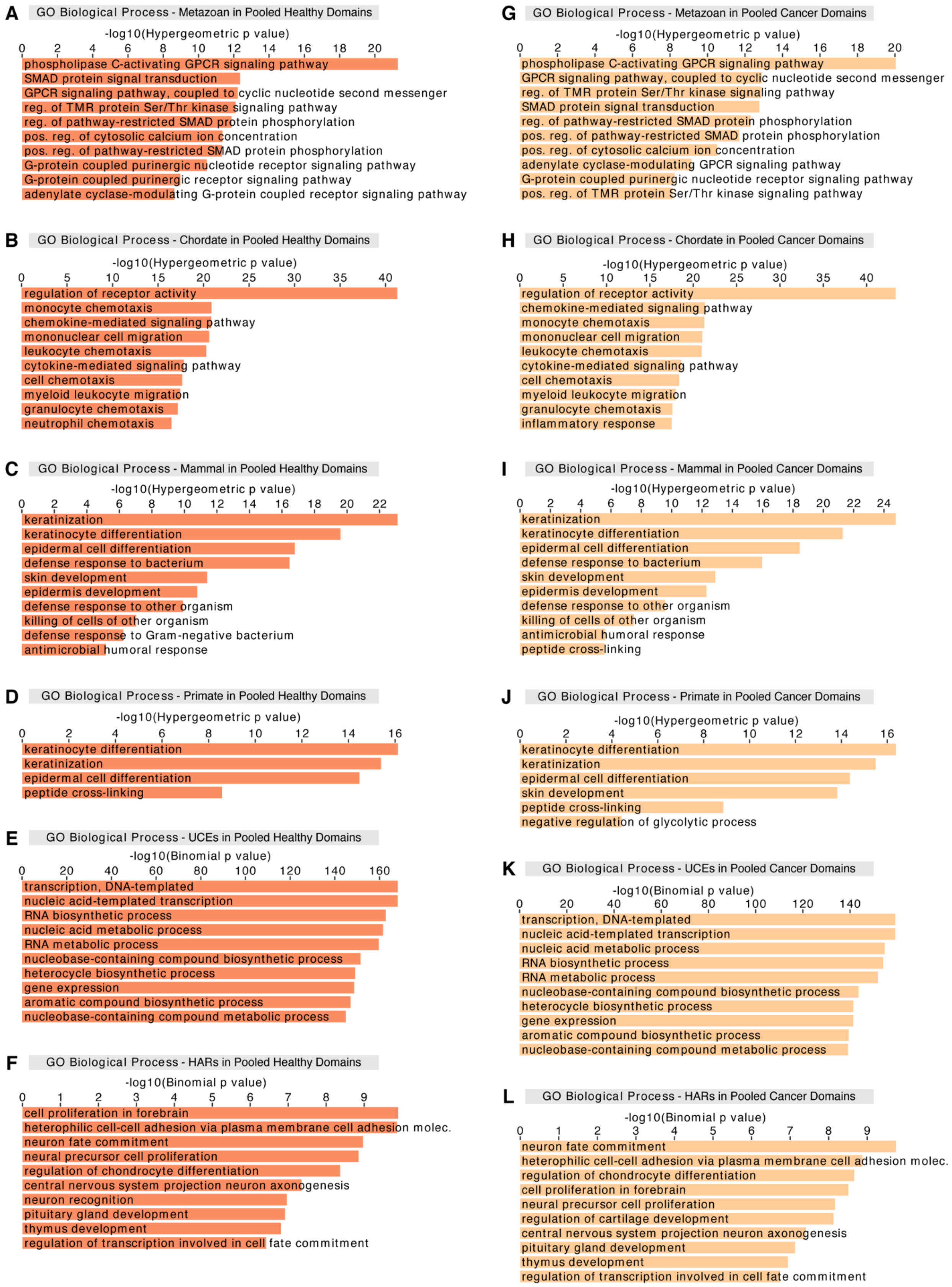

bioRxiv preprint doi: https://doi.org/10.1101/2024.03.17.585403; this version posted November 22, 2024. The copyright holder for this preprint (which was not certified by peer review) is the author/funder, who has granted bioRxiv a license to display the preprint in perpetuity. It is made available under aCC-BY 4.0 International license.

Figure S7. Functional associations of era genes in pooled domains in cancer state are very similar to those in all of the pooled domains and pooled healthy domains.

(A-F) Era genes, UCEs, and HARs intersected with pooled domains in healthy state are associated with nearly identical processes as with all pooled domains (Figures 3, S6K, and S6L). (G-L) When intersected with pooled cancer domains, GO terms are very similar as those in all pooled domains (Figures 3, S6K, and S6L) and pooled healthy domains, with the exception of gained association with the glycolytic process in primate genes (J). GREAT analysis for regions of varying sequence conservation that overlap pooled healthy (A-F) and cancer (G-L) domains including (A and G) metazoan genes, (B and H) chordate genes, (C and I) mammal genes, (D and J) primate genes, (E and K) UCEs, and (F and L) HARs. Backgrounds: all era genes for individual eras of genes; whole genome for UCEs and HARs. Abbreviated GO terms: GPCR, G-protein coupled receptor; reg., regulation; TMR, transmembrane receptor; Ser, serine; Thr, threonine; pos., positive; molec., molecules. Dark orange, pooled healthy domains; light orange, pooled cancer domains.

bioRxiv preprint doi: https://doi.org/10.1101/2024.03.17.585403; this version posted November 22, 2024. The copyright holder for this preprint (which was not certified by peer review) is the author/funder, who has granted bioRxiv a license to display the preprint in perpetuity. It is made available under aCC-BY 4.0 International license.

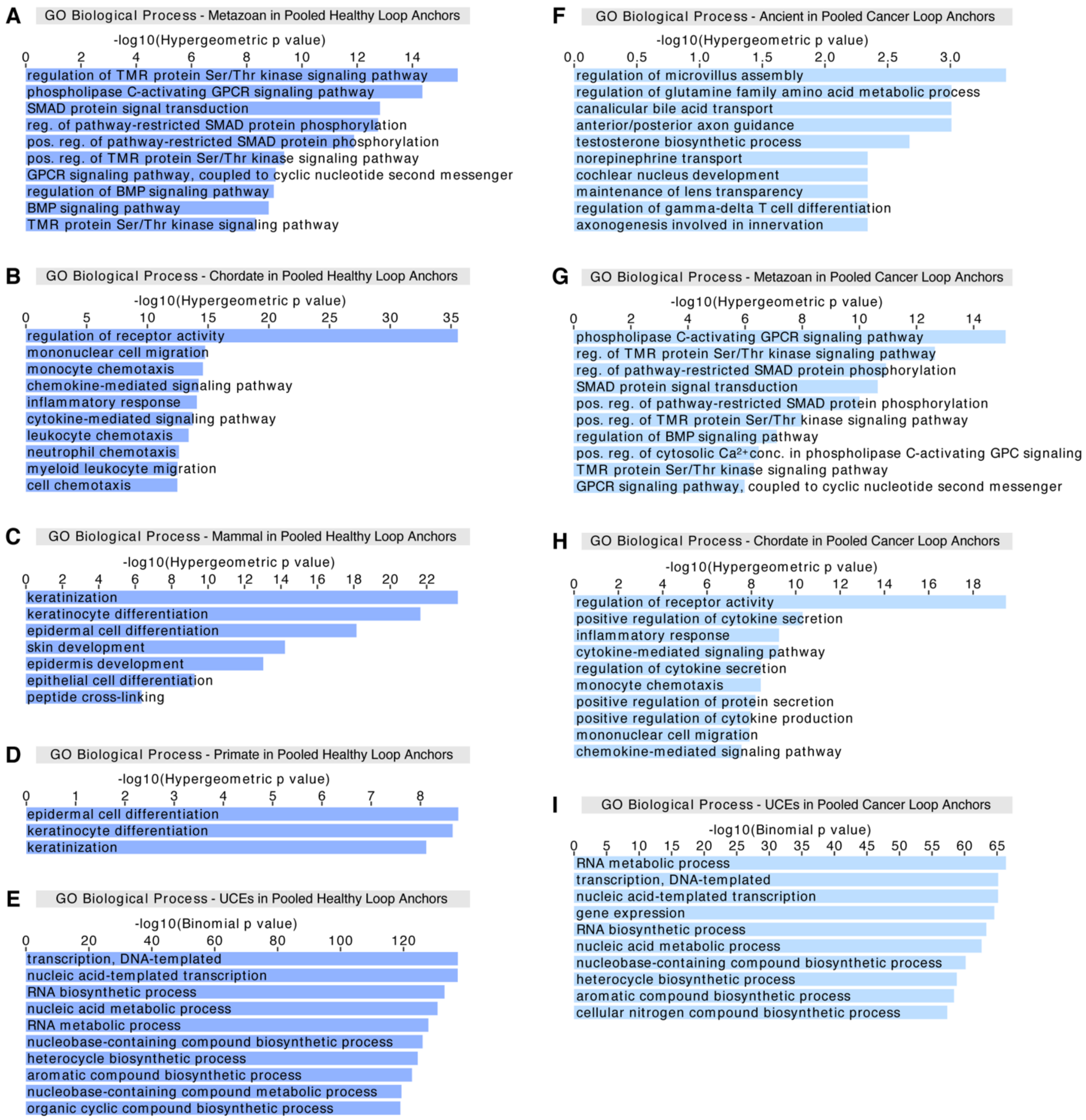

Figure S8. Evolutionarily older genes and UCEs exhibit GO term enrichments when in pooled cancer loop anchors.

In pooled loop anchors in healthy state (dark blue), GO terms are associated with (A) signaling pathways for metazoan genes, (B) immune response-related processes for chordate genes, (C) skin development for mammal genes, (D) keratinocyte differentiation for primate genes, and (E)

bioRxiv preprint doi: https://doi.org/10.1101/2024.03.17.585403; this version posted November 22, 2024. The copyright holder for this preprint (which was not certified by peer review) is the author/funder, who has granted bioRxiv a license to display the preprint in perpetuity. It is made available under aCC-BY 4.0 International license.

transcription and nucleic acid metabolism for UCEs. These GO terms are largely similar to those seen with all pooled loop anchors (Figures S6A-S6E). In pooled cancer loop anchors (light blue), GO terms are related to (F) a wide variety of processes for ancient genes, (G) signaling pathways for metazoan genes, (H) immune response-related processes for chordate genes, and (I) transcription and nucleic acid metabolism for UCEs. Note the absence of GO terms for mammal and primate genes. Backgrounds: all era genes for individual eras of genes; whole genome for UCEs. Abbreviated GO terms: TMR, transmembrane receptor; Ser, serine; Thr, threonine; GPCR, G-protein coupled receptor; reg., regulation; pos., positive; conc., concentration.

bioRxiv preprint doi: https://doi.org/10.1101/2024.03.17.585403; this version posted November 22, 2024. The copyright holder for this preprint (which was not certified by peer review) is the author/funder, who has granted bioRxiv a license to display the preprint in perpetuity. It is made available under aCC-BY 4.0 International license.

Table S1. Information on datasets.

(A) Information on era genes. (B) Control dataset for Igen ORF. (C) Control dataset for Igen Non-ORF. (D) Difference between genes ages estimated as lineage restriction levels by sequence similarity (SeqSim) versus synteny (Synt). (E) Hi-C datasets and related sources.

Table S2. Spearman correlation analyses of the relationships between regions of varying sequence conservation and 3D genomic features (domains, loop anchors, boundaries).

(A) Spearman correlation coefficients using 50-kb bins. (B) p values using 50-kb bins. Non-significant p values (red). (C) Spearman correlation coefficients using 20-kb bins. (D) p values using 20-kb bins. Non-significant p values (red). (E) Spearman correlation coefficients using 100-kb bins. (F) p values using 100-kb bins. Non-significant p values (red).

Table S3. Era genes and gene expression with taxonomic subdivision equivalence between NCBI nodes and GenTree branches.

(A) Mean counts of RNA transcripts in 6 adult human tissue categories in sequences from 7 evolutionary categories. There are 7 evolutionary categories of sequences: 5 evolutionary eras (Ancient, Metazoan, Chordate, Mammal, Primate), Igen ORFs, and Igen Non-ORFs. For every sequence, counts of RNA transcripts obtained using the GTEx database are shown, together with information about protein sequence, genomic localization, and gene evolutionary age. (B) Significance of statistical comparisons of mean counts from different tissue categories and evolutionary categories. Statistical significance was calculated with the Mann-Whitney U test and

bioRxiv preprint doi: https://doi.org/10.1101/2024.03.17.585403; this version posted November 22, 2024. The copyright holder for this preprint (which was not certified by peer review) is the author/funder, who has granted bioRxiv a license to display the preprint in perpetuity. It is made available under aCC-BY 4.0 International license.

corrected for multiple comparisons using the Benjamini-Hochberg correction. (C) Counts of RNA TPM in 6 adult human tissue categories, in sequences from 7 evolutionary categories. As in (A), for every sequence, TPM counts from the GTEx database are shown, together with information about protein sequence, genomic localization, and gene evolutionary age. (D) Comparison of the expression of genes grouped by ages determined by sequence similarity versus synteny. Significance of statistical comparisons of mean counts from different tissue categories and evolutionary categories. Statistical significance was calculated with the Mann-Whitney U test and corrected for multiple comparisons using the Benjamini-Hochberg correction. (E) Significance of statistical comparisons of TPMs from different tissue categories and evolutionary categories. Statistical significance was calculated with the Mann-Whitney U test and corrected for multiple comparisons using the Benjamini-Hochberg correction. (F) Taxonomic subdivision equivalence between NCBI nodes (phylostrata, PS) and GenTree branches. The four evolutionary eras used for the comparison between Sequence Similarity and Synteny (Early, Vertebrate, Mammal, Primate) are indicated to the right. (G) For each protein-coding gene present in the GenTree database and in our Ensembl-derived database, the estimated evolutionary age corresponds to the taxonomic subdivision to which the gene belongs. The taxonomic subdivision is indicated as NCBI nodes (phyostrata, PS) and as GenTree branches. For the comparison between Sequence Similarity and Synteny, four evolutionary eras were used (Early, Vertebrate, Mammal, Primate) to accommodate the GenTree branches. For comparing counts between tissues, five evolutionary eras were used (Ancient, Metazoan, Chordate, Mammal, Primate). For each gene, indicated are the protein length, the amino acid sequence, and the differences between the age determined by synteny versus by sequence similarity.

bioRxiv preprint doi: https://doi.org/10.1101/2024.03.17.585403; this version posted November 22, 2024. The copyright holder for this preprint (which was not certified by peer review) is the author/funder, who has granted bioRxiv a license to display the preprint in perpetuity. It is made available under aCC-BY 4.0 International license.

Supplemental References

1. Shao, Y., Chen, C., Shen, H., He, B.Z., Yu, D., Jiang, S., Zhao, S., Gao, Z., Zhu, Z., Chen, X., et al. (2019). GenTree, an integrated resource for analyzing the evolution and function of primate-specific coding genes. Genome Res *29*, 682-696. 10.1101/gr.238733.118.
2. Consortium, G.T., Laboratory, D.A., Coordinating Center -Analysis Working, G., Statistical Methods groups-Analysis Working, G., Enhancing, G.g., Fund, N.I.H.C., Nih/Nci, Nih/Nhgri, Nih/Nimh, Nih/Nida, et al. (2017). Genetic effects on gene expression across human tissues. Nature *550*, 204-213. 10.1038/nature24277.
