# Supplementary material for: Functional associations of evolutionarily recent human genes exhibit sensitivity to the 3D genome landscape and disease
Source: bioRxiv. 2024 Mar 17:2024.03.17.585403. Preprint. [Version 1] doi: 10.1101/2024.03.17.585403 (PMC10980080; doi:10.1101/2024.03.17.585403)
Supplement: Supplementary file 1 [file NIHPP2024.03.17.585403v1-supplement-1.pdf]

bioRxiv preprint doi: https://doi.org/10.1101/2024.03.17.585403; this version posted March 17, 2024. The copyright holder for this preprint (which was not certified by peer review) is the author/funder, who has granted bioRxiv a license to display the preprint in perpetuity. It is made available under aCC-BY 4.0 International license.

Supporting Information for

Functional associations of evolutionarily recent human genes exhibit sensitivity to the 3D genome landscape and disease

Katherine Fleck[1,2*], Victor Luria[3,4,5,6*], Nitanta Garag[1], Amir Karger[7], Trevor Hunter[1], Daniel Marten[4,5], William Phu[4,5], Kee-Myoung Nam[8], Nenad Sestan[3], Anne H. O'Donnell-Luria[4,5,9#], Jelena Erceg[1,2,10#]

[1]Department of Molecular and Cell Biology, University of Connecticut, Storrs, CT 06269

[2]Institute for Systems Genomics, University of Connecticut, Storrs, CT 06269

[3]Department of Neuroscience, Yale School of Medicine, New Haven, CT 06510

[4]Division of Genetics and Genomics, Boston Children's Hospital, Boston, MA 02115

[5]Program in Medical and Population Genetics, Broad Institute of Harvard and MIT, Cambridge, MA 02142

[6]Department of Systems Biology, Harvard Medical School, Boston, MA 02115

[7]IT-Research Computing, Harvard Medical School, Boston, MA 02115

bioRxiv preprint doi: https://doi.org/10.1101/2024.03.17.585403; this version posted March 17, 2024. The copyright holder for this preprint (which was not certified by peer review) is the author/funder, who has granted bioRxiv a license to display the preprint in perpetuity. It is made available under aCC-BY 4.0 International license.

[8]Department of Molecular, Cellular and Developmental Biology, Yale University, New Haven, CT 06510

[9]Department of Pediatrics, Harvard Medical School, Boston, MA 02115

[10]Department of Genetics and Genome Sciences, University of Connecticut Health Center, Farmington, CT 06030

[*]K.F. and V.L. contributed equally to this work.

[#]To whom correspondence may be addressed. Email: odonnell@broadinstitute.org or jelena.erceg@uconn.edu.

bioRxiv preprint doi: https://doi.org/10.1101/2024.03.17.585403; this version posted March 17, 2024. The copyright holder for this preprint (which was not certified by peer review) is the author/funder, who has granted bioRxiv a license to display the preprint in perpetuity. It is made available under aCC-BY 4.0 International license.

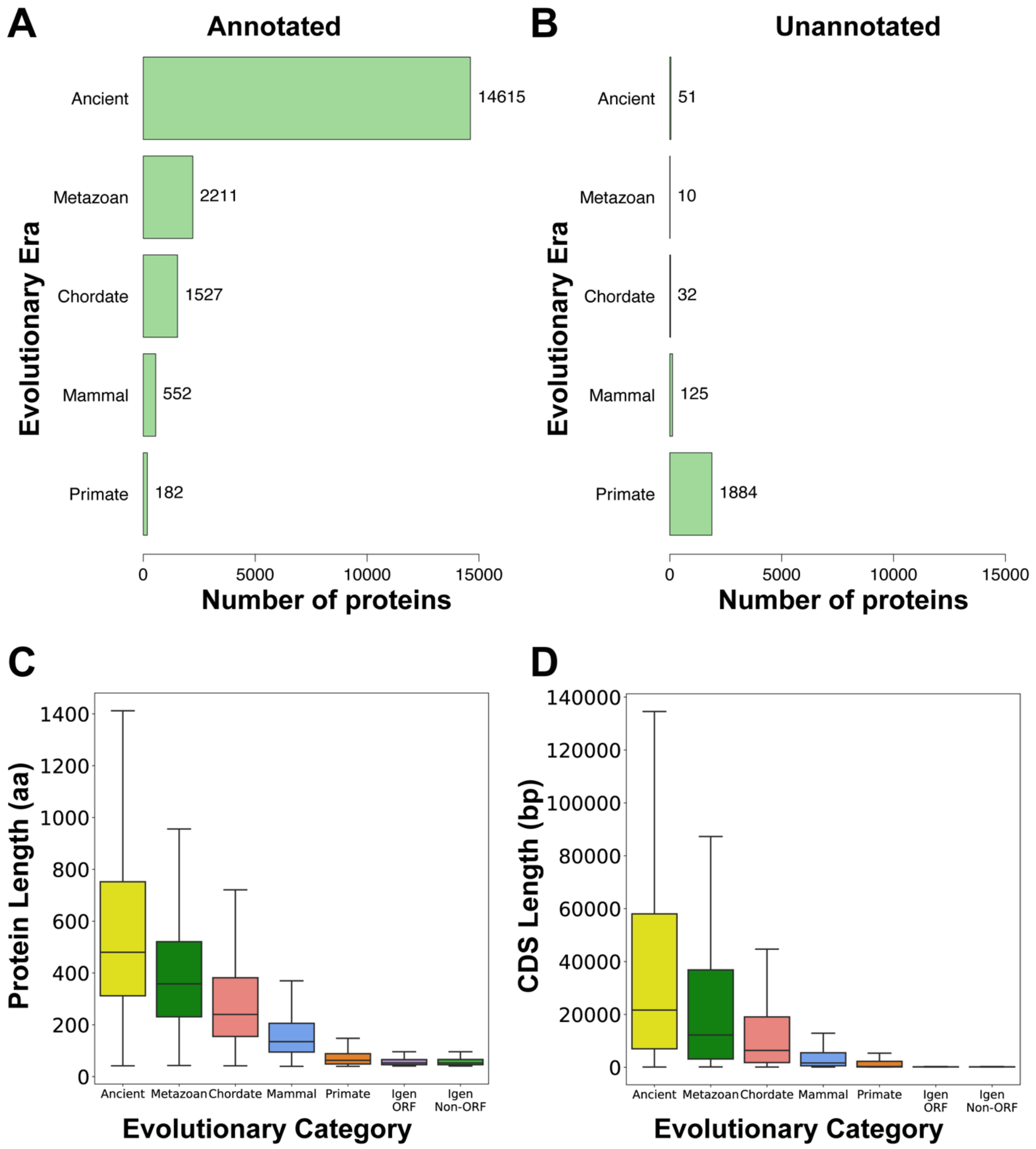

Fig. S1. Gene numbers and lengths for each evolutionary era.

(*A*) Number of single-isoform annotated human protein-coding genes in every evolutionary era (ancient, metazoan, chordate, mammal, primate). Most annotated genes are evolutionarily ancient.

bioRxiv preprint doi: https://doi.org/10.1101/2024.03.17.585403; this version posted March 17, 2024. The copyright holder for this preprint (which was not certified by peer review) is the author/funder, who has granted bioRxiv a license to display the preprint in perpetuity. It is made available under aCC-BY 4.0 International license.

(*B*) Number of single-isoform unannotated human protein-coding genes in every evolutionary era (ancient, metazoan, chordate, mammal, primate). Most unannotated genes are evolutionarily young. (*C*) Protein length is indicated in number of amino acids (AA). The evolutionarily youngest genes are much shorter than ancient genes and only slightly longer than control Igen ORF and Igen Non-ORF sequences. (*D*) The length of coding sequences (CDS) is indicated as the number of base pairs (bp). The evolutionarily youngest genes are shorter than ancient genes and similar to control Igen ORF and Igen Non-ORF sequences.

bioRxiv preprint doi: https://doi.org/10.1101/2024.03.17.585403; this version posted March 17, 2024. The copyright holder for this preprint (which was not certified by peer review) is the author/funder, who has granted bioRxiv a license to display the preprint in perpetuity. It is made available under aCC-BY 4.0 International license.

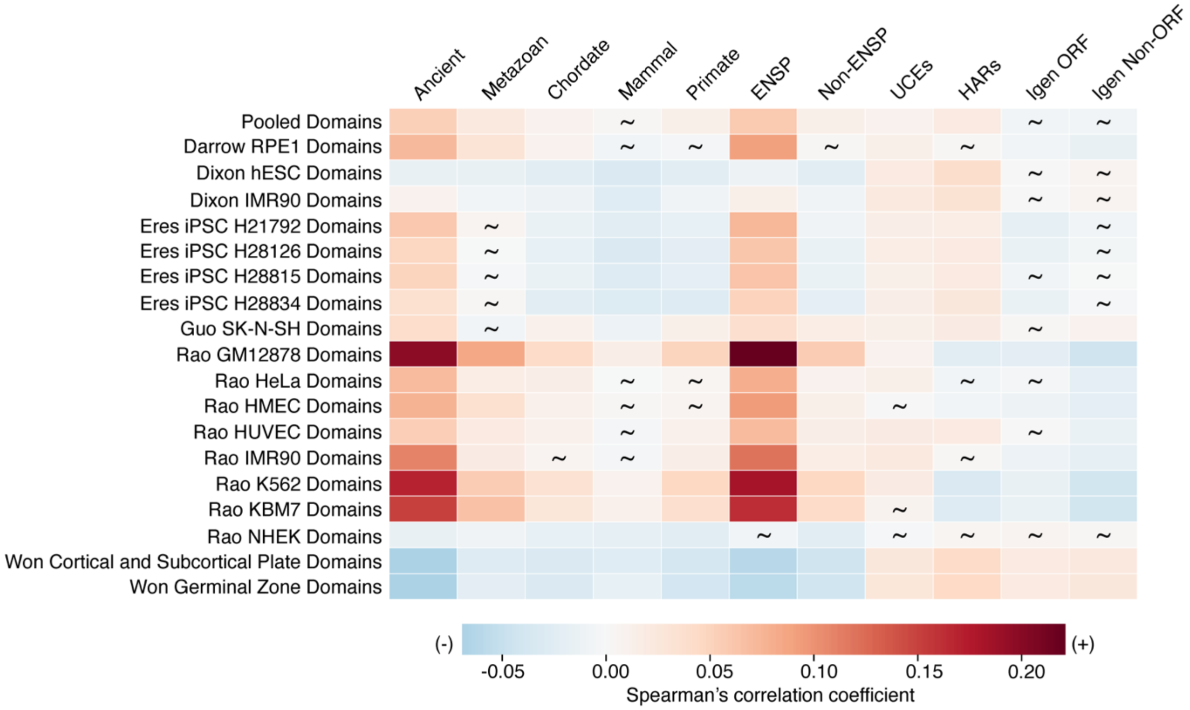

Fig. S2. The relationships between domains and various evolutionary genomic regions show stability or variation across cell types based on sequence conservation.

Ancient genes, annotated ENSP genes, and UCEs are primarily significantly positively correlated with domains ($0 \leq p \leq 0.746$). Conversely, less conserved genes and fast-evolving HARs show variable relationships with domains across cell types ($2.34\times10^{-92} \leq p \leq 0.988$). Spearman correlation analysis was performed by partitioning the genome into 50-kb bins. Spearman correlation coefficients are indicated by a heatmap. Non-significant p values are depicted by tildes. Control datasets include ENSP genes, Non-ENSP genes, Igen ORFs, and Igen Non-ORFs.

bioRxiv preprint doi: https://doi.org/10.1101/2024.03.17.585403; this version posted March 17, 2024. The copyright holder for this preprint (which was not certified by peer review) is the author/funder, who has granted bioRxiv a license to display the preprint in perpetuity. It is made available under aCC-BY 4.0 International license.

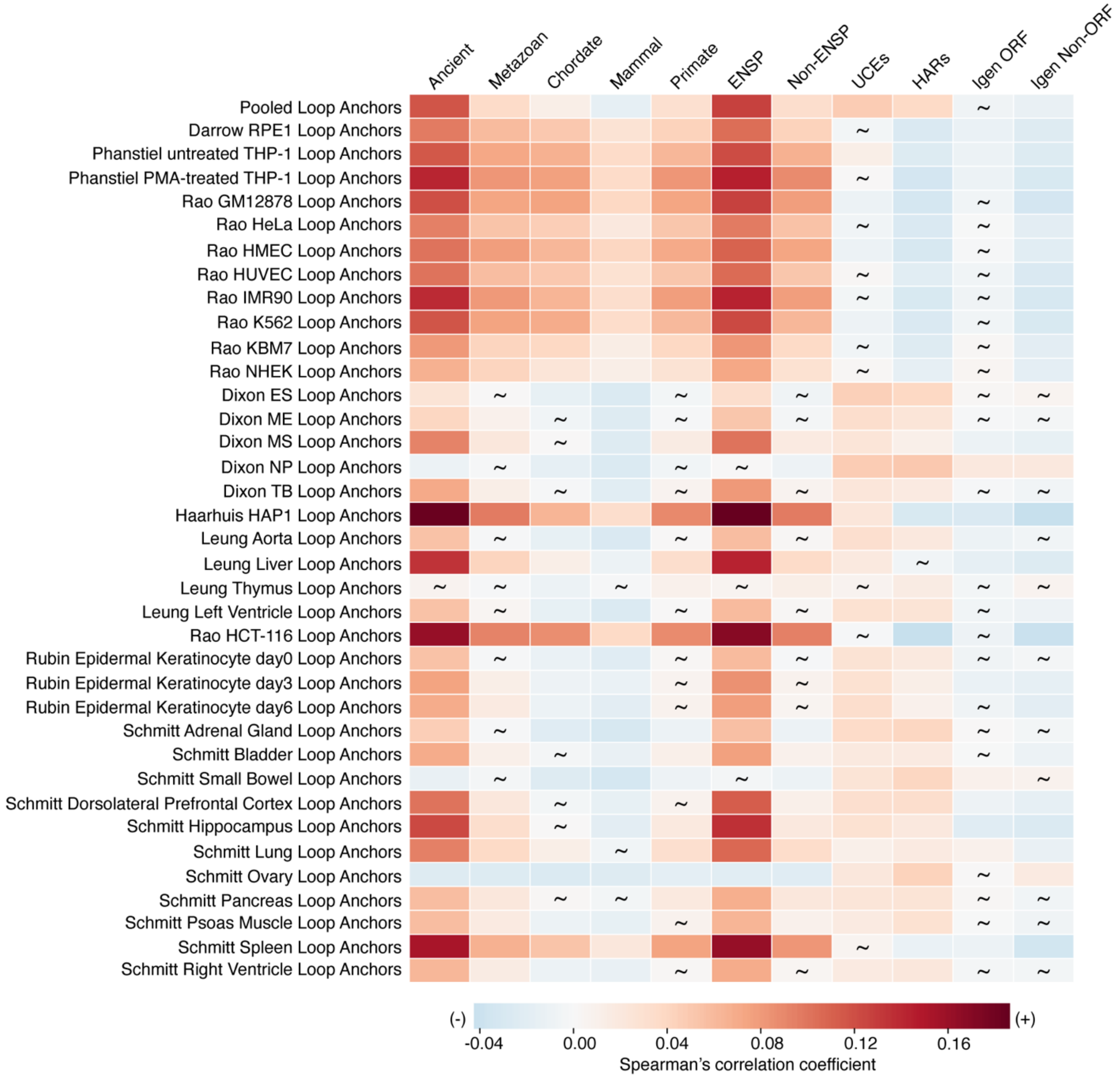

Fig. S3. The relationships between loop anchors and regions of various sequence conservation show different variation levels across cell types.

Ancient and annotated ENSP genes have more consistent relationships with loop anchors by cell type ($0 \leq p \leq 0.944$), whereas less conserved genes, HARs, and UCEs have more variable relationships ($8.65\times10^{-118} \leq p \leq 0.965$). Spearman correlation analysis was performed by partitioning the genome into 50-kb bins. Spearman correlation coefficients are indicated by a

bioRxiv preprint doi: https://doi.org/10.1101/2024.03.17.585403; this version posted March 17, 2024. The copyright holder for this preprint (which was not certified by peer review) is the author/funder, who has granted bioRxiv a license to display the preprint in perpetuity. It is made available under aCC-BY 4.0 International license.

heatmap. Non-significant p values are depicted by tildes. Control datasets include ENSP genes, Non-ENSP genes, Igen ORFs, and Igen Non-ORFs.

bioRxiv preprint doi: https://doi.org/10.1101/2024.03.17.585403; this version posted March 17, 2024. The copyright holder for this preprint (which was not certified by peer review) is the author/funder, who has granted bioRxiv a license to display the preprint in perpetuity. It is made available under aCC-BY 4.0 International license.

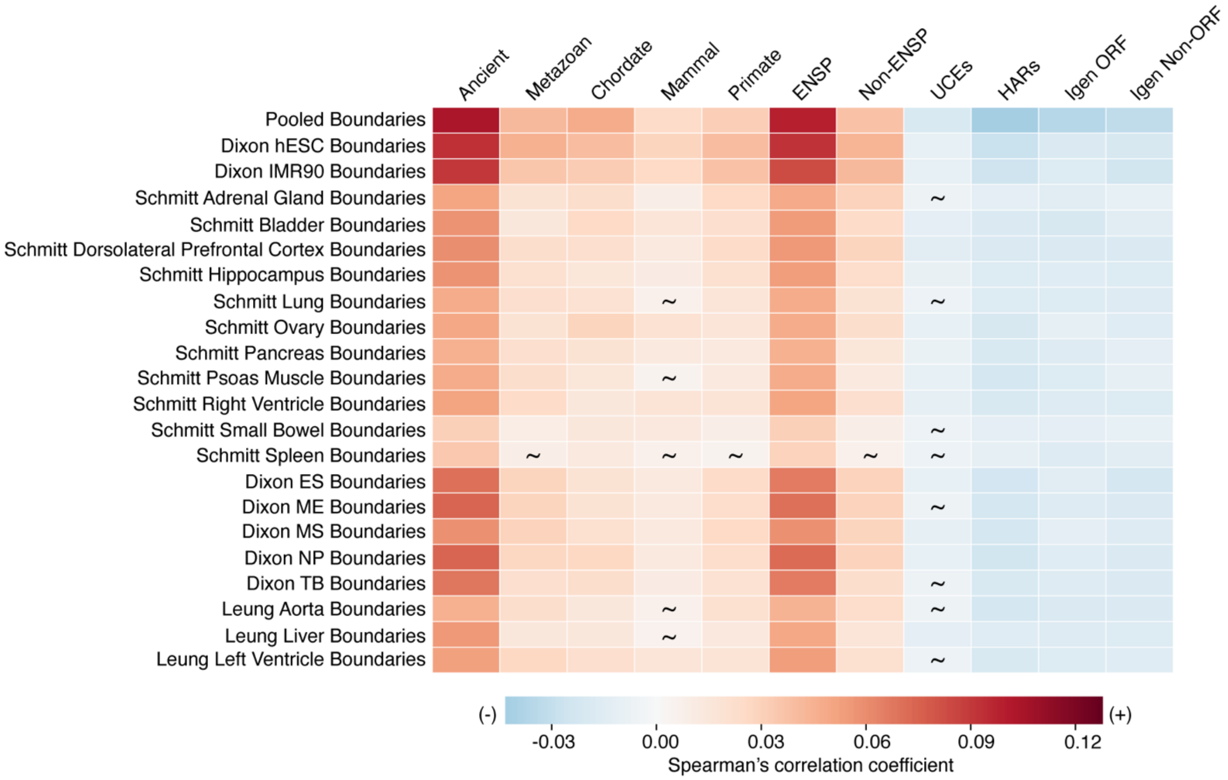

Fig. S4. The relationship of boundaries with era genes, UCEs, and HARs is highly consistent across cell types.

Era genes are predominantly significantly positively correlated with boundaries ($2.19\times10^{-110} \le p \le 0.306$), while UCEs and HARs are non-significantly or significantly negatively correlated ($8.91\times10^{-12} \le p \le 0.136$). Spearman correlation analysis was performed by partitioning the genome into 50-kb bins. Spearman correlation coefficients are indicated by a heatmap. Non-significant p values are depicted by tildes. Control datasets include ENSP genes, Non-ENSP genes, Igen ORFs, and Igen Non-ORFs.

bioRxiv preprint doi: https://doi.org/10.1101/2024.03.17.585403; this version posted March 17, 2024. The copyright holder for this preprint (which was not certified by peer review) is the author/funder, who has granted bioRxiv a license to display the preprint in perpetuity. It is made available under aCC-BY 4.0 International license.

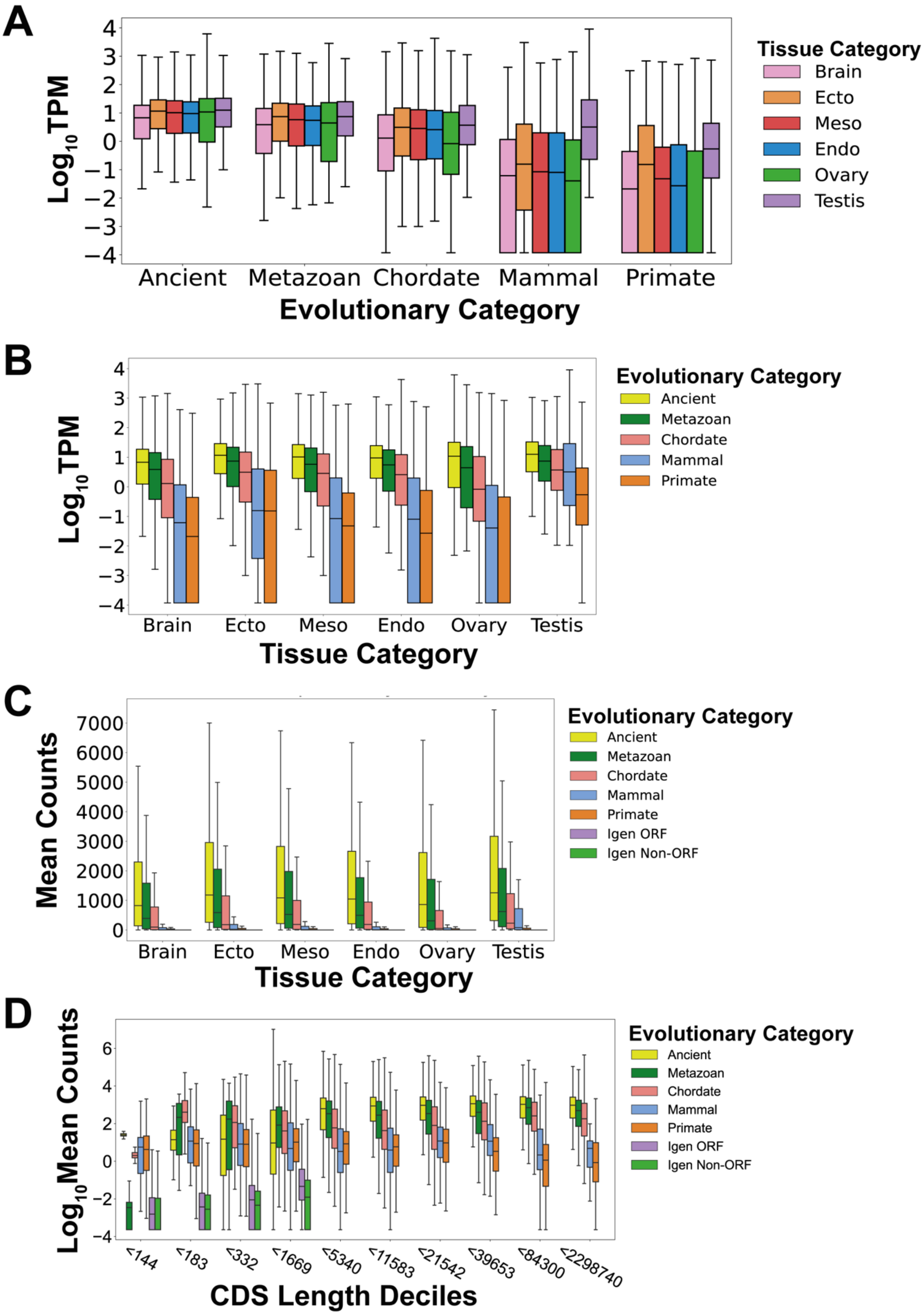

bioRxiv preprint doi: https://doi.org/10.1101/2024.03.17.585403; this version posted March 17, 2024. The copyright holder for this preprint (which was not certified by peer review) is the author/funder, who has granted bioRxiv a license to display the preprint in perpetuity. It is made available under aCC-BY 4.0 International license.

Fig. S5. Transcriptional level variation with gene age, tissue of origin and gene length.

As in Fig. 2, the expression of human protein-coding genes is measured for RNA transcripts across 54 human tissues from the GTEx database (1). (*A*) RNA expression levels, measured as log10 of TPMs, increase from youngest to oldest genes (Table S7). Within mammal and primate genes, expression in testis is higher than in other tissues. (*B*) RNA expression levels, measured as log10 of TPMs, in 6 tissue categories increase from youngest genes to oldest genes. (*C*) The raw mean counts highlight that expression is highest in testis and lowest in brain. (*D*) RNA expression levels, measured as log10 of mean counts, increase with the gene length, shown as CDS length. Within 7 CDS length deciles, ancient genes are expressed at highest levels while primate genes are expressed at lowest levels. Within 3 of the shorter CDS length deciles, the highest expression is that of metazoan genes or chordate genes. Only the first 4 deciles have control non-genic sequences, and their expression is 100-1000 times lower than that of genes.

bioRxiv preprint doi: https://doi.org/10.1101/2024.03.17.585403; this version posted March 17, 2024. The copyright holder for this preprint (which was not certified by peer review) is the author/funder, who has granted bioRxiv a license to display the preprint in perpetuity. It is made available under aCC-BY 4.0 International license.

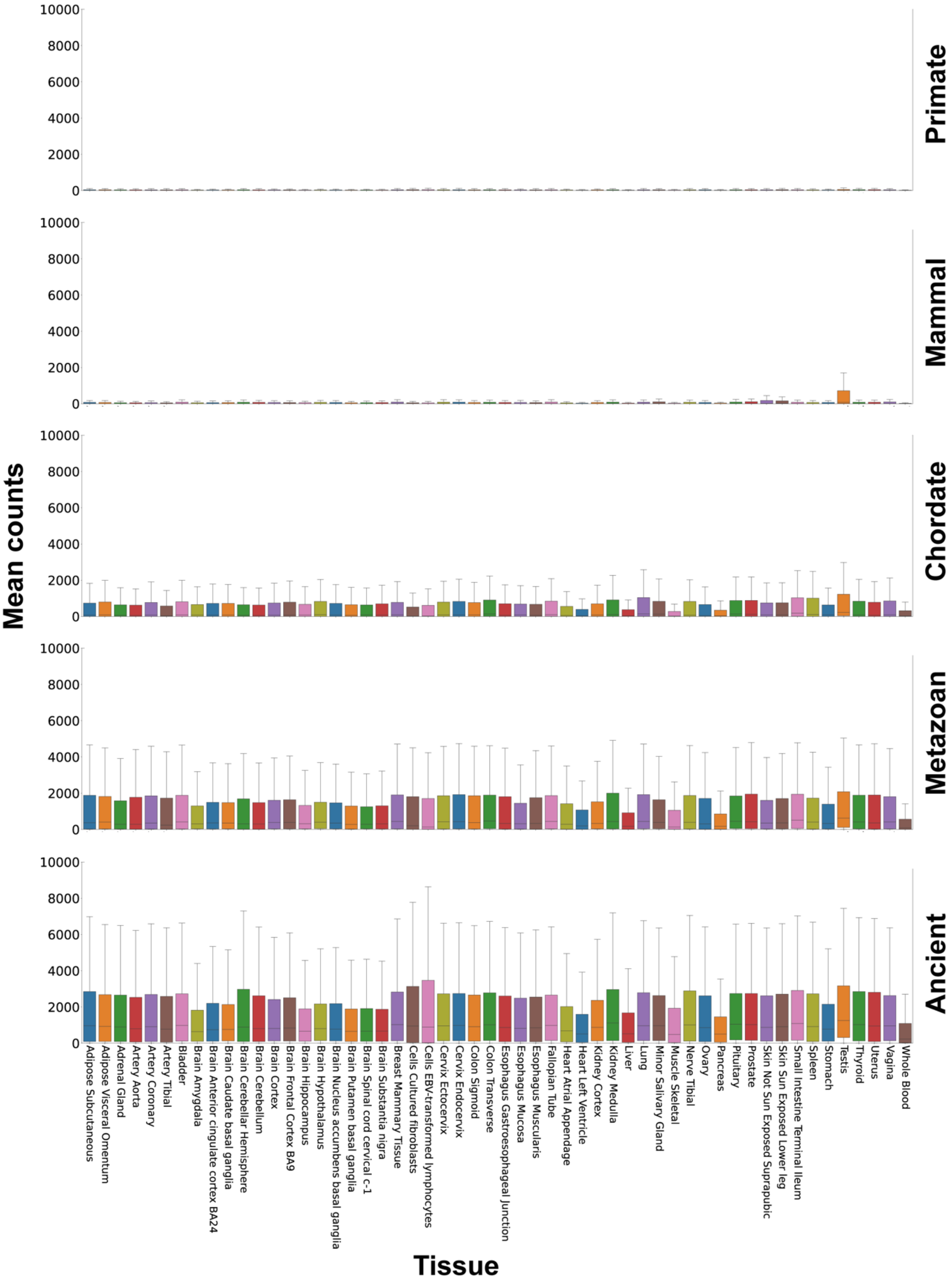

bioRxiv preprint doi: https://doi.org/10.1101/2024.03.17.585403; this version posted March 17, 2024. The copyright holder for this preprint (which was not certified by peer review) is the author/funder, who has granted bioRxiv a license to display the preprint in perpetuity. It is made available under aCC-BY 4.0 International license.

Fig. S6. Gene expression in 54 human tissues and evolutionary eras.

Gene expression levels are measured as mean counts of RNA transcripts across 54 human tissues in 5 evolutionary eras, as in Fig. 2 and Fig. S5. Gene expression increases evolutionarily from primate to ancient genes. With each evolutionary era, testis expression is typically highest, followed by other tissues which differ between eras.

bioRxiv preprint doi: https://doi.org/10.1101/2024.03.17.585403; this version posted March 17, 2024. The copyright holder for this preprint (which was not certified by peer review) is the author/funder, who has granted bioRxiv a license to display the preprint in perpetuity. It is made available under aCC-BY 4.0 International license.

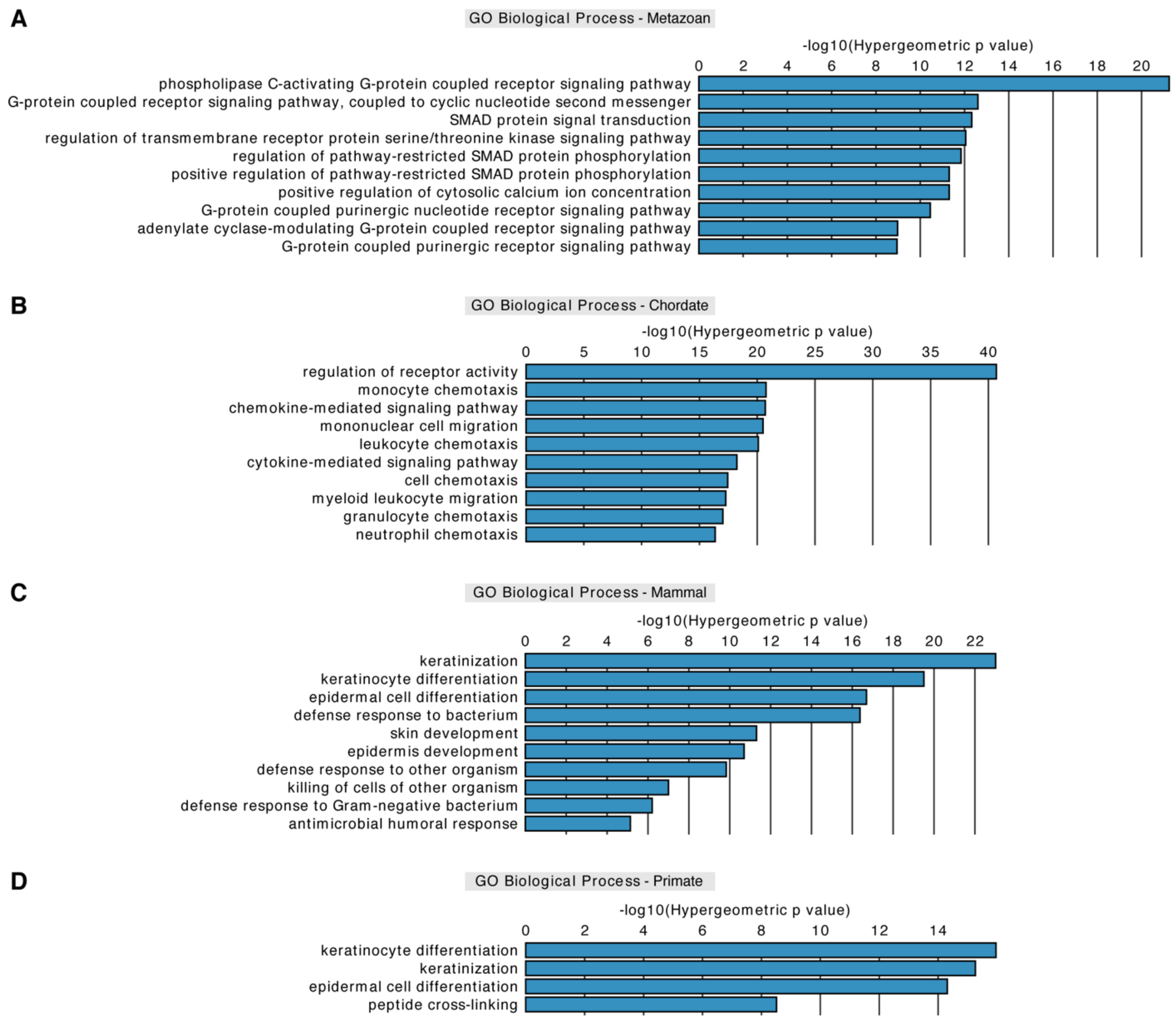

Fig. S7. Functional associations of era genes.

GREAT analysis for (*A*) metazoan genes, (*B*) chordate genes, (*C*) mammal genes, and (*D*) primate genes. Those GO terms are associated with very similar processes as for era genes in pooled domains (Fig. 3). (*A*-*D*) Analysis performed against all era genes as a background.

bioRxiv preprint doi: https://doi.org/10.1101/2024.03.17.585403; this version posted March 17, 2024. The copyright holder for this preprint (which was not certified by peer review) is the author/funder, who has granted bioRxiv a license to display the preprint in perpetuity. It is made available under aCC-BY 4.0 International license.

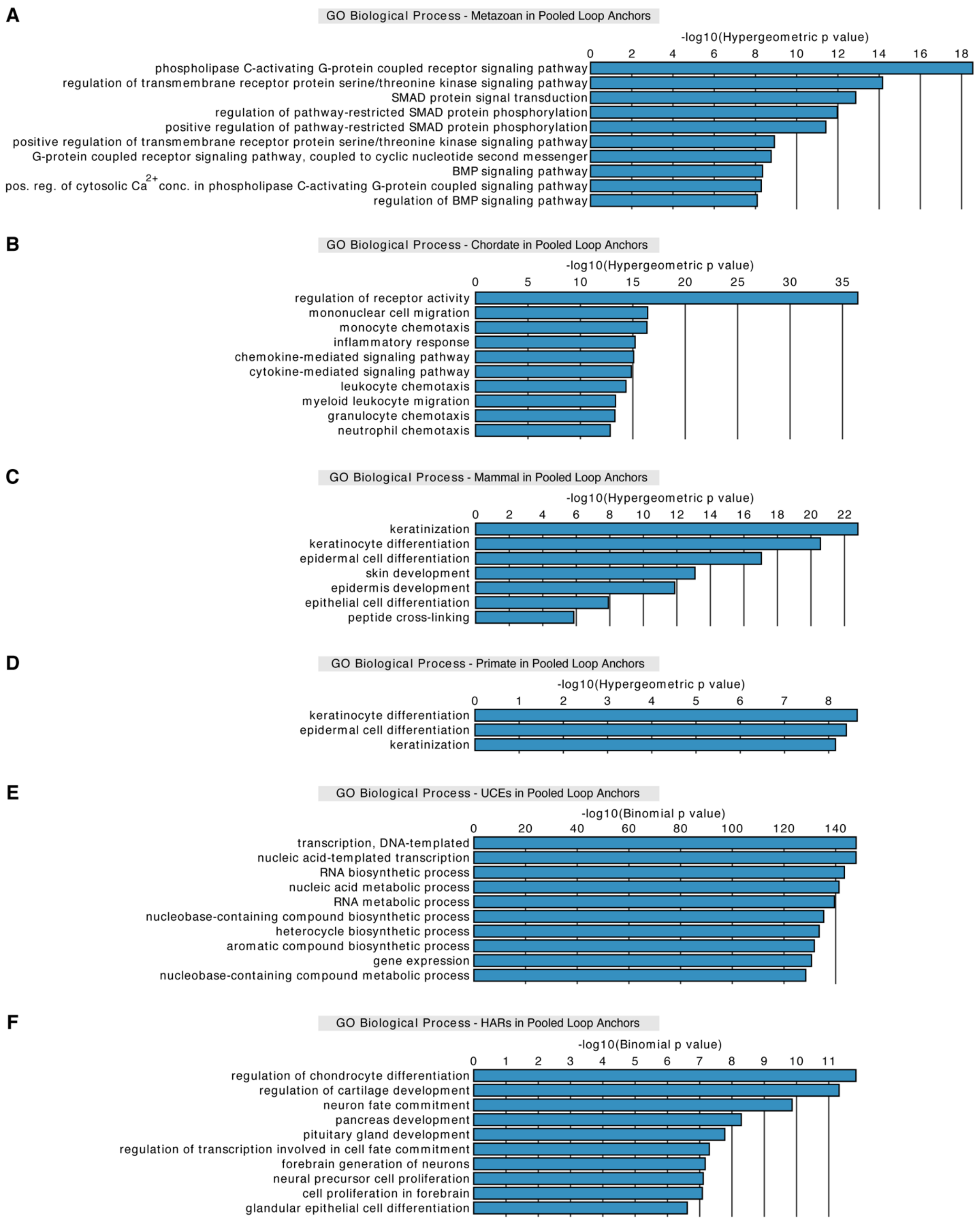

Fig. S8. Functional associations of era genes, UCEs, and HARs in pooled loop anchors.

bioRxiv preprint doi: https://doi.org/10.1101/2024.03.17.585403; this version posted March 17, 2024. The copyright holder for this preprint (which was not certified by peer review) is the author/funder, who has granted bioRxiv a license to display the preprint in perpetuity. It is made available under aCC-BY 4.0 International license.

Regions of varying sequence conservation overlapping pooled loop anchors are linked to GO terms associated with (*A*) signaling pathways for metazoan genes, (*B*) immune response-related processes for chordate genes, (*C*) skin development for mammal genes (note absence of defense response compared to Fig. 3*C*), (*D*) keratinocyte differentiation for primate genes (note absence of peptide cross-linking compared to Fig. 3*D*), (*E*) transcription and RNA metabolic processes for UCEs, and (*F*) neuronal processes, cartilage formation, pancreas development, and glandular epithelial cell differentiation for HARs. Backgrounds: all era genes for individual eras of genes; whole genome for UCEs and HARs.

bioRxiv preprint doi: https://doi.org/10.1101/2024.03.17.585403; this version posted March 17, 2024. The copyright holder for this preprint (which was not certified by peer review) is the author/funder, who has granted bioRxiv a license to display the preprint in perpetuity. It is made available under aCC-BY 4.0 International license.

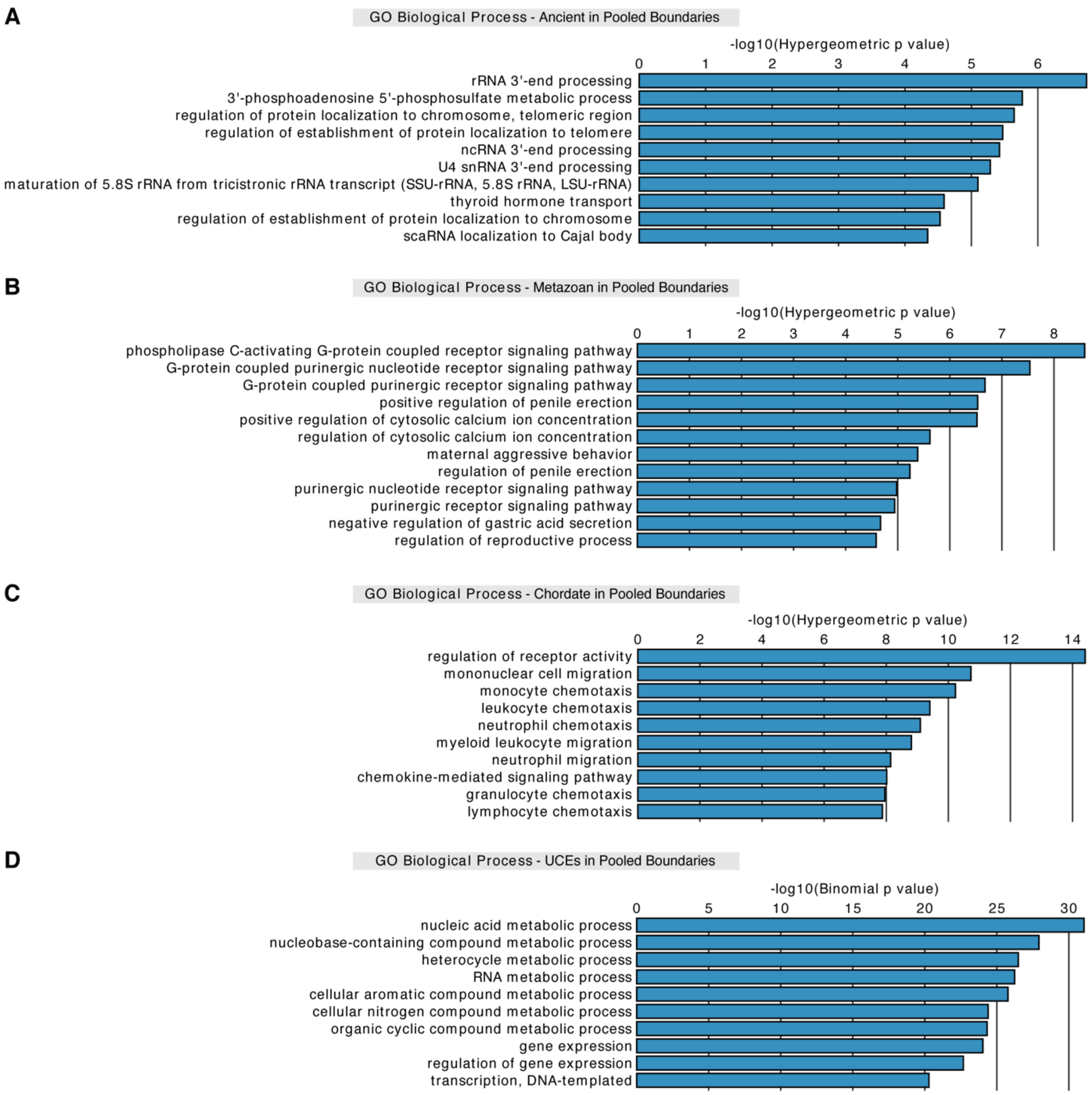

Fig. S9. Functional associations of era genes and UCEs in pooled boundaries.

In pooled boundaries, enrichments for GO terms are related to (*A*) protein localization to telomeres and ncRNA processes for ancient genes, (*B*) signaling pathways and regulation of reproductive processes for metazoan genes, (*C*) immune response-related processes for chordate genes, and (*D*)

bioRxiv preprint doi: https://doi.org/10.1101/2024.03.17.585403; this version posted March 17, 2024. The copyright holder for this preprint (which was not certified by peer review) is the author/funder, who has granted bioRxiv a license to display the preprint in perpetuity. It is made available under aCC-BY 4.0 International license.

transcription and RNA metabolic processes for UCEs. Backgrounds: all era genes for individual eras of genes; whole genome for UCEs.

bioRxiv preprint doi: https://doi.org/10.1101/2024.03.17.585403; this version posted March 17, 2024. The copyright holder for this preprint (which was not certified by peer review) is the author/funder, who has granted bioRxiv a license to display the preprint in perpetuity. It is made available under aCC-BY 4.0 International license.

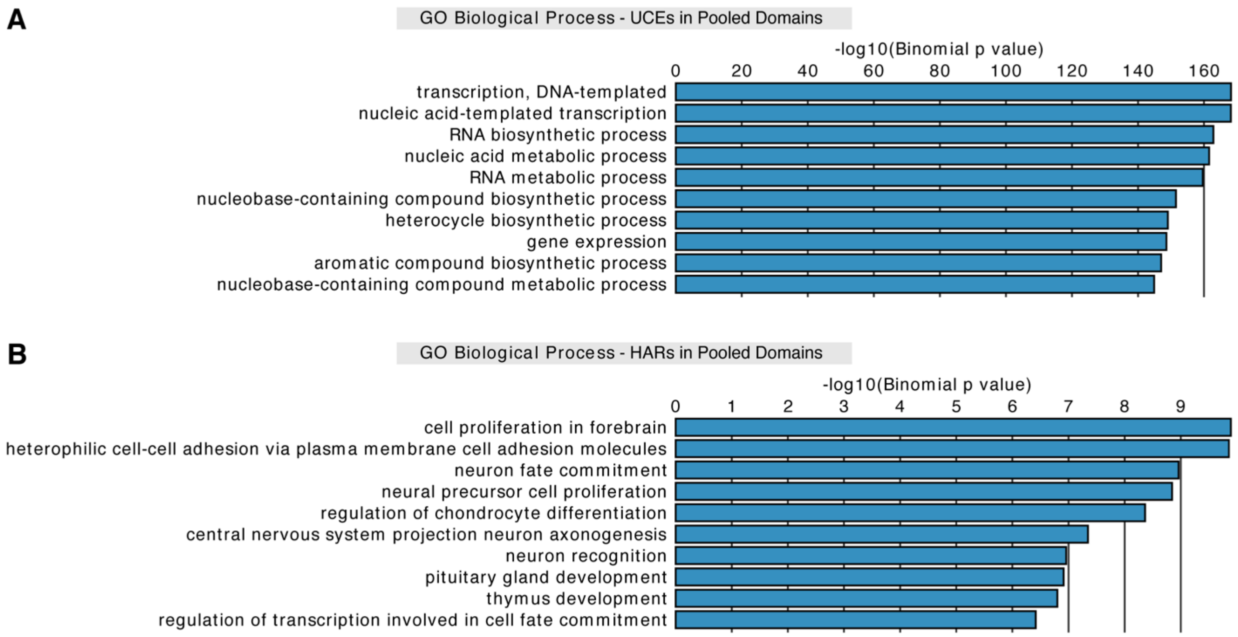

Fig. S10. Functional associations of UCEs and HARs in pooled domains.

GREAT analysis for UCEs and HARs that overlap pooled domains. (*A*) UCEs in pooled domains are associated with transcription and nucleic acid metabolism, and (*B*) HARs in pooled domains are associated with neuronal processes, cartilage formation, and thymus development. Background: whole genome.

bioRxiv preprint doi: https://doi.org/10.1101/2024.03.17.585403; this version posted March 17, 2024. The copyright holder for this preprint (which was not certified by peer review) is the author/funder, who has granted bioRxiv a license to display the preprint in perpetuity. It is made available under aCC-BY 4.0 International license.

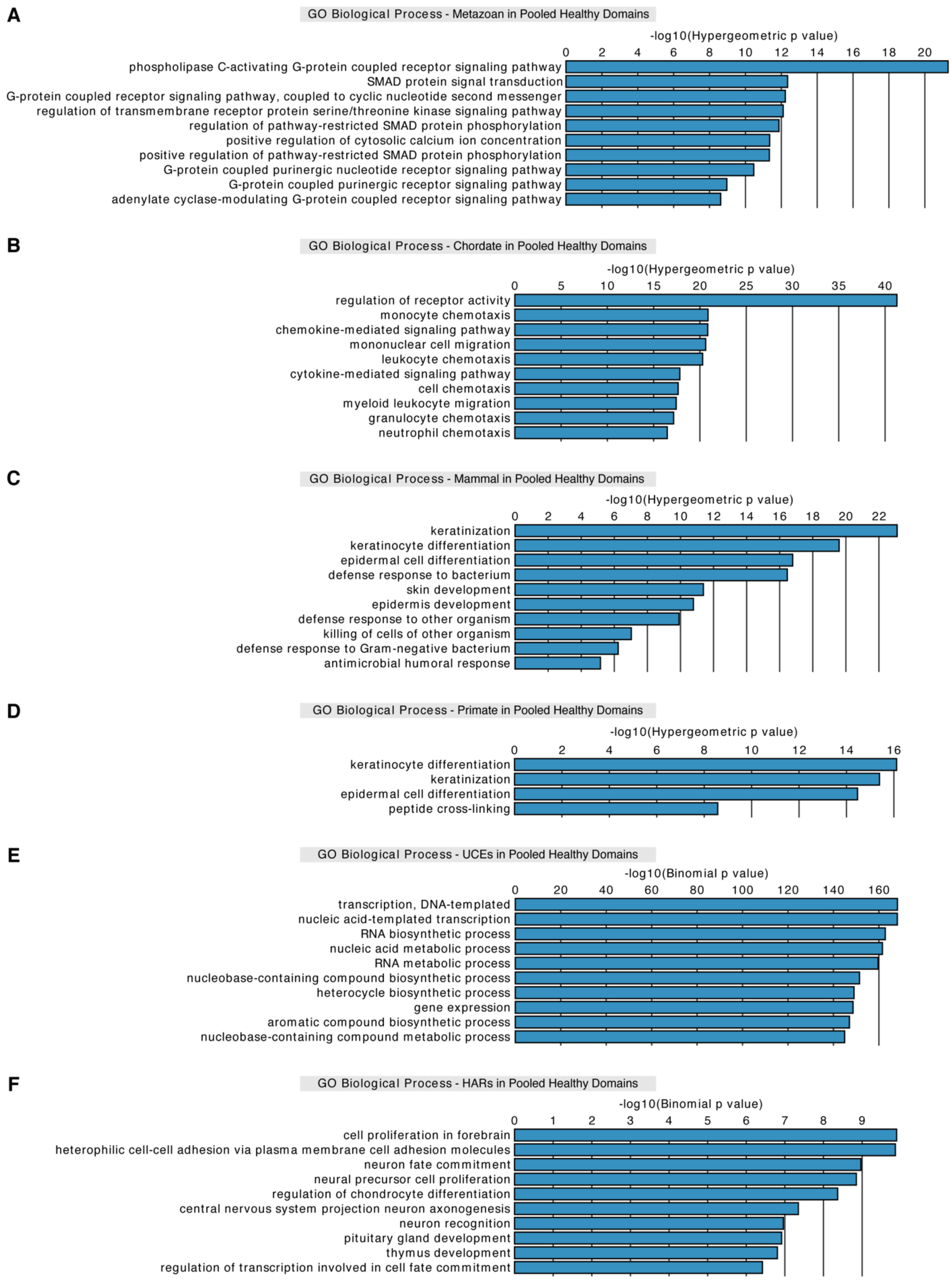

bioRxiv preprint doi: https://doi.org/10.1101/2024.03.17.585403; this version posted March 17, 2024. The copyright holder for this preprint (which was not certified by peer review) is the author/funder, who has granted bioRxiv a license to display the preprint in perpetuity. It is made available under aCC-BY 4.0 International license.

Fig. S11. Era genes intersected with pooled domains in healthy state are associated with nearly identical processes as with all pooled domains.

GREAT analysis for regions of varying sequence conservation that overlap pooled healthy domains including (*A*) metazoan genes, (*B*) chordate genes, (*C*) mammal genes, (*D*) primate genes, (*E*) UCEs, and (*F*) HARs. Enrichments for GO terms are related to nearly identical processes as for era genes, UCEs, and HARs in all of the pooled domains (Fig. 3 and Fig. S10). Backgrounds: all era genes for individual eras of genes; whole genome for UCEs and HARs.

bioRxiv preprint doi: https://doi.org/10.1101/2024.03.17.585403; this version posted March 17, 2024. The copyright holder for this preprint (which was not certified by peer review) is the author/funder, who has granted bioRxiv a license to display the preprint in perpetuity. It is made available under aCC-BY 4.0 International license.

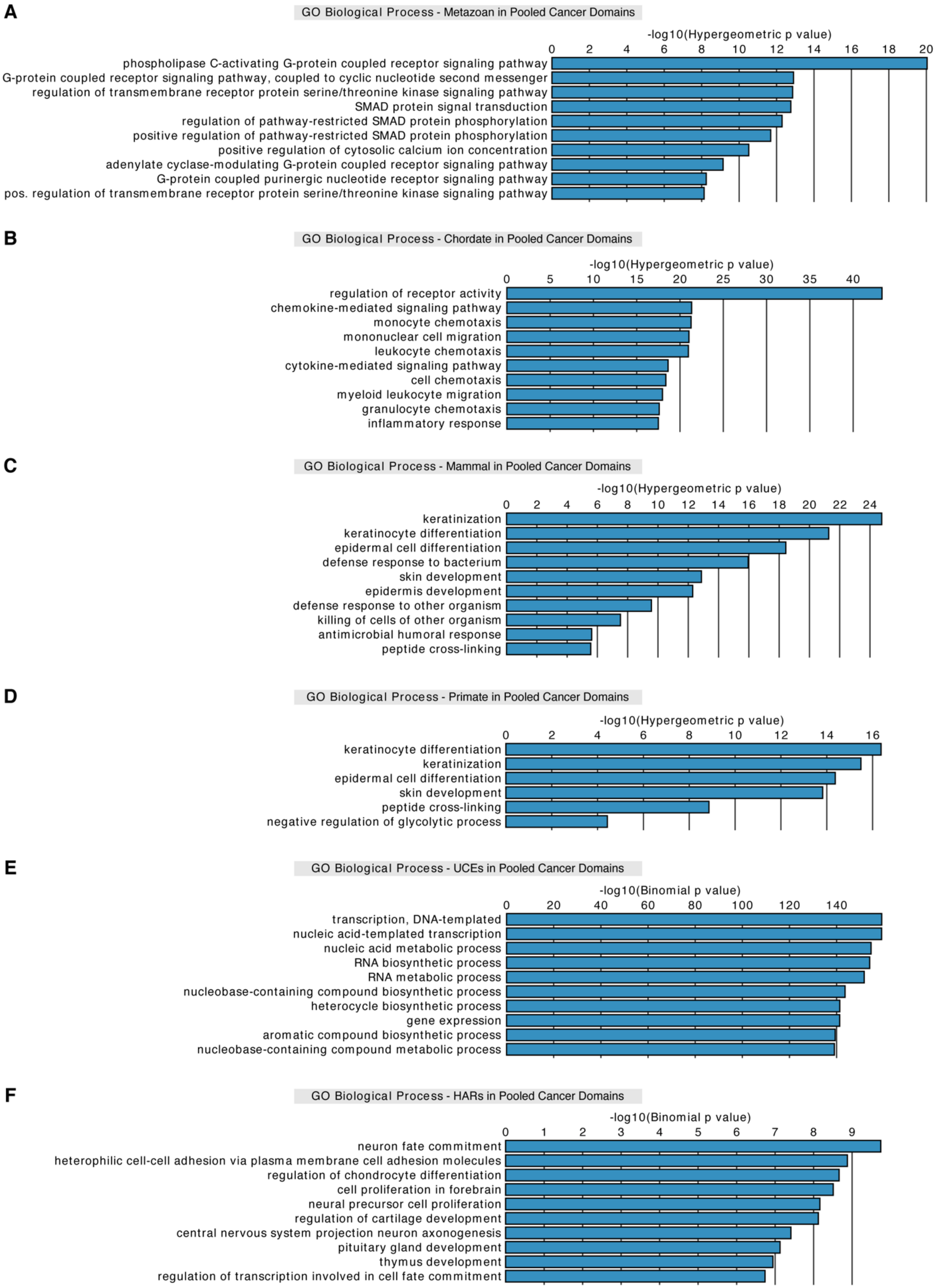

bioRxiv preprint doi: https://doi.org/10.1101/2024.03.17.585403; this version posted March 17, 2024. The copyright holder for this preprint (which was not certified by peer review) is the author/funder, who has granted bioRxiv a license to display the preprint in perpetuity. It is made available under aCC-BY 4.0 International license.

Fig. S12. Functional associations of era genes in pooled domains in cancer state are very similar to those in all of the pooled domains and pooled healthy domains.

When intersected with pooled cancer domains, different regions of varying sequence conservation including (*A*) metazoan genes, (*B*) chordate genes, (*C*) mammal genes, (*D*) primate genes, (*E*) UCEs, and (*F*) HARs are associated with very similar GO terms as those in all pooled domains (Fig. 3 and Fig. S10) and pooled healthy domains (Fig. S11), with the exception of gained association with the glycolytic process in primate genes (*D*). Backgrounds: all era genes for individual eras of genes; whole genome for UCEs and HARs.

bioRxiv preprint doi: https://doi.org/10.1101/2024.03.17.585403; this version posted March 17, 2024. The copyright holder for this preprint (which was not certified by peer review) is the author/funder, who has granted bioRxiv a license to display the preprint in perpetuity. It is made available under aCC-BY 4.0 International license.

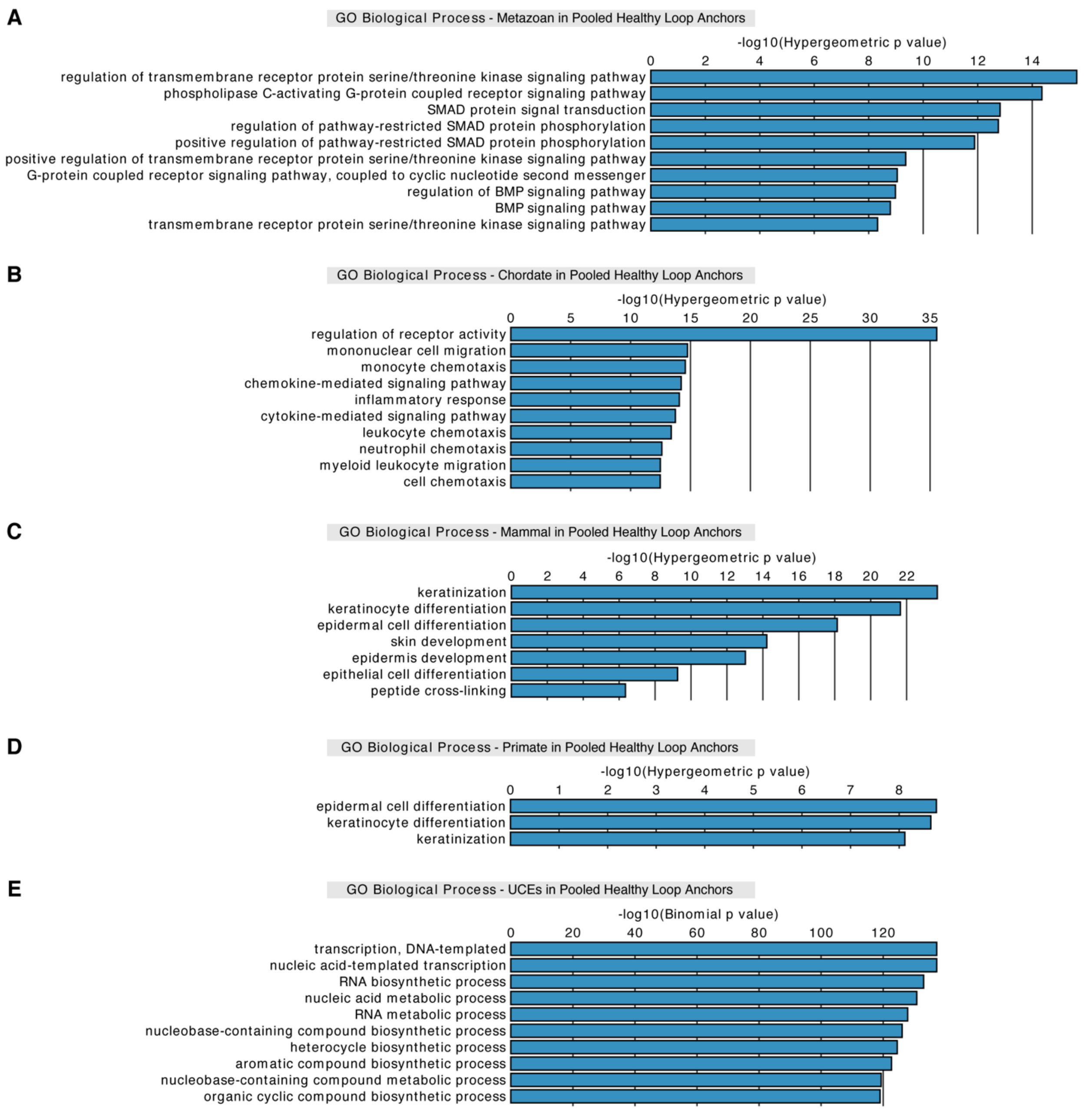

Fig. S13. Functional associations of era genes and UCEs intersected with pooled loop anchors in healthy state closely resemble those in all pooled loop anchors.

In pooled loop anchors in healthy state, GO terms are associated with (*A*) signaling pathways for metazoan genes, (*B*) immune response-related processes for chordate genes, (*C*) skin development for mammal genes, (*D*) keratinocyte differentiation for primate genes, and (*E*) transcription and

bioRxiv preprint doi: https://doi.org/10.1101/2024.03.17.585403; this version posted March 17, 2024. The copyright holder for this preprint (which was not certified by peer review) is the author/funder, who has granted bioRxiv a license to display the preprint in perpetuity. It is made available under aCC-BY 4.0 International license.

nucleic acid metabolism for UCEs. These GO terms are largely similar to those seen with all pooled loop anchors (Fig. S8). Backgrounds: all era genes for individual eras of genes; whole genome for UCEs.

bioRxiv preprint doi: https://doi.org/10.1101/2024.03.17.585403; this version posted March 17, 2024. The copyright holder for this preprint (which was not certified by peer review) is the author/funder, who has granted bioRxiv a license to display the preprint in perpetuity. It is made available under aCC-BY 4.0 International license.

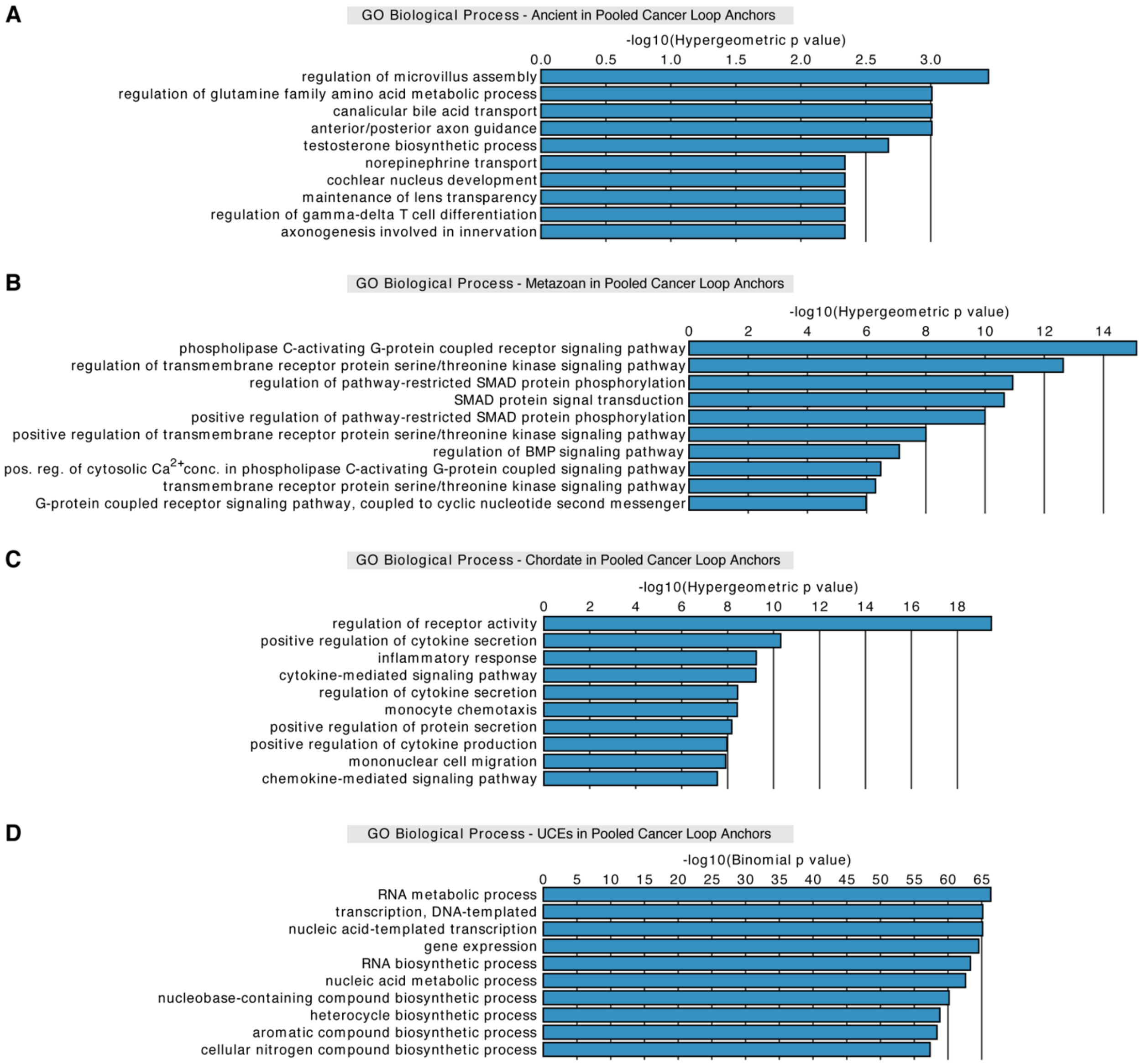

Fig. S14. Evolutionarily older genes and UCEs exhibit GO term enrichments when in pooled cancer loop anchors.

In pooled cancer loop anchors, GO terms are related to (*A*) a wide variety of processes for ancient genes, (*B*) signaling pathways for metazoan genes, (*C*) immune response-related processes for chordate genes, and (*D*) transcription and nucleic acid metabolism for UCEs. Note the absence of

bioRxiv preprint doi: https://doi.org/10.1101/2024.03.17.585403; this version posted March 17, 2024. The copyright holder for this preprint (which was not certified by peer review) is the author/funder, who has granted bioRxiv a license to display the preprint in perpetuity. It is made available under aCC-BY 4.0 International license.

GO terms for mammal and primate genes. Backgrounds: all era genes for individual eras of genes; whole genome for UCEs.

bioRxiv preprint doi: https://doi.org/10.1101/2024.03.17.585403; this version posted March 17, 2024. The copyright holder for this preprint (which was not certified by peer review) is the author/funder, who has granted bioRxiv a license to display the preprint in perpetuity. It is made available under aCC-BY 4.0 International license.

Table S1. Information on datasets.

(*A*) Information on era genes. (*B*) Control dataset for Igen ORF. (*C*) Control dataset for Igen Non-ORF. (*D*) Hi-C datasets and related sources.

Table S2. Spearman correlation analysis of the relationship between regions of varying sequence conservation and 3D genomic features (domains, loop anchors, boundaries) by partitioning the genome into 50-kb bins.

(*A*) Spearman correlation coefficients using 50-kb bins. (*B*) p values using 50-kb bins. Non-significant p values (red).

Table S3. Spearman correlation analysis of the relationship between regions of varying sequence conservation and 3D genomic features (domains, loop anchors, boundaries) by partitioning the genome into 20-kb bins.

(*A*) Spearman correlation coefficients using 20-kb bins. (*B*) p values using 20-kb bins. Non-significant p values (red).

Table S4. Spearman correlation analysis of the relationship between regions of varying sequence conservation and 3D genomic features (domains, loop anchors, boundaries) by partitioning the genome into 100-kb bins.

bioRxiv preprint doi: https://doi.org/10.1101/2024.03.17.585403; this version posted March 17, 2024. The copyright holder for this preprint (which was not certified by peer review) is the author/funder, who has granted bioRxiv a license to display the preprint in perpetuity. It is made available under aCC-BY 4.0 International license.

(*A*) Spearman correlation coefficients using 100-kb bins. (*B*) p values using 100-kb bins. Non-significant p values (red).

Table S5. Mean counts of RNA transcripts in 6 adult human tissue categories in sequences from 7 evolutionary categories. There are 7 evolutionary categories of sequences: 5 evolutionary eras (Ancient, Metazoan, Chordate, Mammal, Primate), Igen ORFs, and Igen Non-ORFs. For every sequence, counts of RNA transcripts obtained using the GTEx database are shown, together with information about protein sequence, genomic localization, and gene evolutionary age.

Table S6. Significance of statistical comparisons of mean counts from different tissue categories and evolutionary categories. Statistical significance was calculated with the Mann-Whitney U test and corrected for multiple comparisons using the Benjamini-Hochberg correction.

Table S7. Counts of RNA TPM in 6 adult human tissue categories, in sequences from 7 evolutionary categories. As in Table S5, for every sequence, TPM counts from the GTEx database are shown, together with information about protein sequence, genomic localization, and gene evolutionary age.

SI References

1. G. T. Consortium *et al*., Genetic effects on gene expression across human tissues. *Nature* **550**, 204-213 (2017).
